# Ethnobotanical Surveys of Plants Used by Quilombola Communities in Brazil: A Scoping Review

**DOI:** 10.3390/life14101215

**Published:** 2024-09-24

**Authors:** Letícia Francine Silva Ramos, Ananda Gomes de Sousa, Rebeca de Siqueira Amorim, Alan de Araújo Roque, Israel Luís Diniz Carvalho, Ana Laura Vilela de Carvalho, Milena Evangelista dos Santos, Maiara Bernardes Marques, Luiza Rayanna Amorim de Lima, Moan Jéfter Fernandes Costa, Pedro Henrique Sette-de-Souza

**Affiliations:** 1 Programa de Pós-Graduação em Saúde e Desenvolvimento Socioambiental, Universidade de Pernambuco, Campus Garanhuns, Garanhuns 55294-902, Pernambuco, Brazilmilena.evangelistasantos@upe.br (M.E.d.S.);; 2 Faculdade de Odontologia, Universidade de Pernambuco, Campus Arcoverde, Arcoverde 56503-146, Pernambuco, Brazil; 3 Herbário do Parque Estadual Dunas do Natal, Natal 59078-970, Rio Grande do Norte, Brazil

**Keywords:** ethnopharmacological knowledge, natural products, therapeutic uses, public health

## Abstract

Quilombola communities play a crucial role in maintaining biodiversity through traditional management models. The use of medicinal plants within these communities reflects a deep reservoir of knowledge, passed down through generations. The objective of this study was to conduct a scoping review to systematically analyze and synthesize the existing literature on the medicinal plants used by Quilombola communities in Brazil, with a focus on their therapeutic applications and cultural significance. The Population, Concept, and Context (PCC) strategy was utilized, where the population refers to the Quilombolas, the concept pertains to medicinal plants, and the context involves illness. A total of 888 studies were initially identified, but only 10 met the inclusion criteria, covering 297 plant species from 80 different families. These plants are employed in a wide range of therapeutic applications, with decoction, alcohol maceration, and infusion being the most common methods of preparation. The study highlights the rich ethnopharmacological knowledge held by Quilombola communities and underscores the need for greater recognition and integration of this traditional knowledge into public health practices. The conclusion emphasizes the importance of preserving and validating the use of medicinal plants by these communities, which could serve as a foundation for future pharmacological discoveries and the development of culturally appropriate health interventions.

## 1. Introduction

Brazil is recognized as the most biodiverse country in the world, containing six biomes that highlight its rich and varied flora [1]. The Ministry of the Environment reports that 46,355 plant species have been documented across the nation [2]. However, Brazil has experienced ongoing environmental degradation, largely due to economic activities that began during the colonial period. This has led to a significant loss of many species, including those with important therapeutic properties [3].

The knowledge of medicinal plant use for therapeutic purposes is deeply rooted in traditional populations, particularly among Quilombola and indigenous communities [4]. Quilombola communities, in particular, play a crucial role in preserving biodiversity through their traditional management practices [5]. Understanding the interactions within these traditional communities is vital for developing public policies that aim to preserve their cultural heritage and support the continuation of their knowledge and practices [6].

For two centuries, Brazil was a major participant in the transatlantic slave trade, standing out for both the large number of enslaved Africans it trafficked and the prolonged duration of this practice within its borders [7]. Enslaved Africans were subjected to various forms of physical, social, and psychological violence, leading many to flee in search of safer spaces, which resulted in the creation of quilombos—fortresses of resistance and cultural preservation [8]. These communities became sanctuaries where the African identity, culture, and knowledge, both ancestral and gained in Brazil, were maintained and reinvented [9]. The intimate connection with nature in these territories ensured the perpetuation of their way of life [10]. However, the lack of legal recognition for Quilombola territories has led to ongoing disputes that expose these communities to social vulnerabilities, such as rural violence and inequities in accessing health services [11]. Stigmatized and marginalized, many Quilombolas feel alienated from broader society and often turn to traditional healers instead of utilizing the limited services available to them [12].

The use of medicinal plants reflects the accumulated knowledge of traditional communities, passed down through generations, primarily through oral tradition. This empirical knowledge is deeply rooted in the lived experiences of these communities [13].

Medicinal plant species hold great significance in these communities, particularly as they emphasize sustainable development [14]. This emphasis on sustainability enhances the living conditions of these populations, as they possess extensive knowledge of plants and methods for treating various ailments. It is, therefore, crucial to integrate scientific and traditional knowledge to strengthen plant conservation practices [14].

Ethnobotanical surveys are conducted to underscore the importance of these plants in the discovery of new medicines and to deepen our understanding of the intricate relationships between communities and their local flora. These surveys also explore the cultural practices and beliefs associated with different plant uses [15,16]. Research on the use of medicinal plants by Quilombola communities is not only relevant but also vital for advancing scientific knowledge, preserving cultural heritage, and promoting sustainable development at both national and international levels.

Recognizing the importance of preserving cultural traditions alongside therapeutic practices involving medicinal plants, it is essential to review the existing literature. This will help systematize knowledge and expand scientific research on the subject. Thus, the objective of this study was to conduct a scoping review on the use of medicinal plants by Quilombola communities in Brazil.

## 2. Materials and Methods

This study followed a combination of two contemporary protocols for conducting scoping reviews: the PRISMA-ScR guidelines [17] and the Joanna Briggs Institute [18]. The final protocol has been registered with the Open Science Framework (osf.io/h4u82). To be included in the study, publications had to meet the following criteria: (i) they must be peer-reviewed; (ii) they must identify, through herbarium specimens, which plants Quilombola communities use to treat health problems; and (iii) they must be written in Portuguese, English, or Spanish. There were no restrictions on the publication date. Excluded materials included reviews, book chapters, books, theses, dissertations, monographs, letters to the editor, and case reports. These eligibility criteria are consistent with the “Joanna Briggs Institute Reviewer’s Manual” [18].

The Population, Concept, and Context (PCC) strategy was employed to determine the key topics [18]. In this framework, “population” referred to the Quilombolas, “concept” to medicinal plants, and “context” to health-related issues. Studies that did not explicitly identify their samples as Quilombolas were excluded, given that public health policies for Quilombolas are based on official recognition of individuals or communities as such.

The bibliographic databases selected for the search were as follows: the Virtual Health Library (VHL), PubMed, EMBASE, Scopus, Web of Science, Science Direct, and SciELO. Additionally, a manual search was conducted using reference lists from Google Scholar to include gray literature and extend the search scope. Metadata from the search databases were exported to the Rayyan QCRI online platform (RRID:SCR_017584) for analysis.

The search algorithm was tailored to each database, using the following Mesh Terms combined with the Boolean operators “OR” and “AND”: (i) “Ethnic Groups” OR “Quilombolas” AND “Plants, medicinal” OR “Phytotherapy” OR “Medicine, traditional” OR “Ethnobotany”; (ii) “Surveys” OR “questionnaires” OR “Ethnic groups” OR “Quilombolas” AND “Medicinal plants” OR “Phytotherapy” OR “Traditional medicine” OR “Ethnobotany”. The search was conducted in both Portuguese and English to maximize the search scope. There was no limitation on the study period, and the geographical scope covered the entire country, as Quilombola communities are found exclusively in Brazil.

Two reviewers independently and blindly assessed all the identified papers. The Rayyan platform was used for this selection process. Initially, duplicates were removed, followed by incomplete abstracts. The remaining papers were then evaluated based on their titles and abstracts, according to the inclusion and exclusion criteria. In cases of a discrepancy, a third reviewer (A.L.V.C.) was consulted. Researchers underwent prior calibration using 177 studies, and the intra-examiner agreement was verified (100% agreement; kappa 1.00, 95% CI; 95.74% agreement; kappa 0.91, 95% CI; 100% agreement; kappa 1.00, 95% CI, for examiners 1, 2, and 3, respectively) as well as inter-observer agreement (96.25% agreement; kappa 0.93, 95% CI).

The extracted information included the study’s characteristics (the authors, year of publication, and objective), research specifics (the location, design, and sample), and details on the plants used (their scientific name, preparation method, and the health condition treated). The main findings were synthesized following the “JBI Evidence Synthesis Manual” guidelines [19].

The results were organized and presented according to the medicinal applications of the plants. All the plants are listed in Table 1, categorized by the cited literature, year of publication, Quilombola community location, plant family, species, common name, part used, and therapeutic indications. After organizing the data, the scientific names of each plant were verified using the Tropicos platform (https://www.tropicos.org/home—accessed on 27 August 2024), and their conservation status was assessed through the Flora and Funga of Brazil 2020 platform (https://floradobrasil.jbrj.gov.br—accessed on 27 August 2024).

In the synthesis of the results, we used the International Classification of Primary Care—ICPC-2 [20] to identify the bodily systems most frequently targeted by medicinal plants in Quilombola communities.

**Table 1:** Summary of ethnopharmacological data on medicinal plants used by Brazilian Quilombola Communities. NR = Not Reported.

Authors	Year of Publication	Localization	Family	Species	Local Name	Used Part	Preparation	Therapeutic Indications
Barboza da Silva et al. [21]	2012	Barra II, Bahia	Adoxaceae	*Sambucus* sp.	Sabugueiro	Leaves; Flower	Decoction; Infusion	Headache; Fever; Inflammation
Barboza da Silva et al. [21]	2012	Barra II, Bahia	Amaranthaceae	*Alternanthera brasiliana* (L.) Kuntze	Anador/bezetacil	Leaves	Infusion	Urine inflammation
Barboza da Silva et al. [21]	2012	Barra II, Bahia	Amaranthaceae	*Celosia* sp.	Crista do galo	Leaves	Decoction; Infusion; Juice	Trauma; Helminthiasis; Chest pain; Toothache; Pereba; Women’s pain; Women’s inflammation; Gastritis; Flu
Barboza da Silva et al. [21]	2012	Barra II, Bahia	Amaranthaceae	*Dysphania ambrosioides* (L.) Mosyakij and Clemants	Mastruz	Whole plant	Decoction; Maceration in Alcohol	Flu; Cough; Bellyache; Inflammation
Barboza da Silva et al. [21]	2012	Barra II, Bahia	Amaryllidaceae	*Allium ascalonicum* L.	Cebola-branca	Bulb	Decoction; Maceration in Alcohol	To avoid stroke; Epilepsy; Cough; Flu; Constipation; Stomach; Throat; Fortification
Barboza da Silva et al. [21]	2012	Barra II, Bahia	Amaryllidaceae	*Allium cepa* L.	Cebola	Bulb	Decoction; Juice; Maceration	Fortification; Flu; Pressure; Amebiasis
Barboza da Silva et al. [21]	2012	Barra II, Bahia	Amaryllidaceae	*Allium sativum* L.	Alho	Bulb	Infusion	Urethra; Bellyache
Barboza da Silva et al. [21]	2012	Barra II, Bahia	Anacardiaceae	*Anacardium occidentale* L.	Caju branco	Leaves	Decoction	Flu; Fever; Women’s disease; Cough
Barboza da Silva et al. [21]	2012	Barra II, Bahia	Anacardiaceae	*Astronium urundeuva* (M.Allemão) Engl.	Aroeira	Leaves	Infusion; Decoction; Maceration	Wound healing; Ulcer; Toothache; Postpartum cleansing; Women’s inflammation; Chest pain
Barboza da Silva et al. [21]	2012	Barra II, Bahia	Anacardiaceae	*Mangifera indica* L.	Manga espada	Leaves	Infusion; Decoction	Blood pressure; Dysentery
Barboza da Silva et al. [21]	2012	Barra II, Bahia	Anacardiaceae	*Spondias purpurea* L.	Seringuela	Leaves	Decoction; Infusion	Cancer; Diabetes; High blood pressure
Barboza da Silva et al. [21]	2012	Barra II, Bahia	Annonaceae	*Annona muricata* L.	Graviola	Leaves; Bark; Fruit	Decoction; Infusion	Stomach pain; Gasses; Dysentery
Barboza da Silva et al. [21]	2012	Barra II, Bahia	Apiaceae	*Foeniculum vulgare* Mill.	Erva doce	Leaves; Fruit	Others	Rheumatism
Barboza da Silva et al. [21]	2012	Barra II, Bahia	Araceae	*Philodendron* sp.	Imbí, imbé	Leaves	Infusion; Decoction	Stomachache; Bellyache
Barboza da Silva et al. [21]	2012	Barra II, Bahia	Asteraceae	*Acanthospermum glabratum* (DC.) Wild.	Carrapicho roxo	Whole plant	Maceration in Alcohol; Infusion; *In Natura*	Headache; Flu
Barboza da Silva et al. [21]	2012	Barra II, Bahia	Asteraceae	*Achillea millefolium* L.	Arcanfor	Leaves	Decoction; Infusion	Flu; Dysentery
Barboza da Silva et al. [21]	2012	Barra II, Bahia	Asteraceae	*Achyrocline satureioides* (Lam.) DC.	Macela do campo	Leaves; Flower	Decoction; Infusion	Body aches; Sight; Hoarseness; Flu; Phlegm
Barboza da Silva et al. [21]	2012	Barra II, Bahia	Asteraceae	*Ageratum conyzoides* L.	Mentrasto	Whole plant	Decoction; Infusion; Maceration in Alcohol	Menstrual cramps; Inflammation; Heart; Stomach; Throat; Fortification
Barboza da Silva et al. [21]	2012	Barra II, Bahia	Asteraceae	*Artemisia* sp.	Losna	Leaves	Juice; Infusion; Maceration in Alcohol	Sight; Postpartum cleansing
Barboza da Silva et al. [21]	2012	Barra II, Bahia	Asteraceae	*Artemisia vulgaris* L.	Artemijo	Leaves	Decoction; Infusion; Maceration in Alcohol	Blood pressure; Urethra pain; Kidney inflammation; Postpartum cleansing; Diabetes
Barboza da Silva et al. [21]	2012	Barra II, Bahia	Asteraceae	*Bidens pilosa* L.	Carrapicho	Seeds; Leaves	Decoction	Back pain; Women’s inflammation; High blood pressure; Kidney
Barboza da Silva et al. [21]	2012	Barra II, Bahia	Asteraceae	*Chamaecrista blanchetti* (Benth.) Conc., L.P. Queiroz and G.P. Lewis	Rompe gibão	Leaves	Decoction; Infusion; Maceration in Alcohol	Bellyache; Fever; Phlegm; Flu; Menstrual cramps; Nausea; Postpartum cleansing
Barboza da Silva et al. [21]	2012	Barra II, Bahia	Asteraceae	*Gymnanthemum amygdalium* (Delile) Sch.Bip. *ex* Walp.	Aluão	Leaves	Others	To avoid stroke; Headache
Barboza da Silva et al. [21]	2012	Barra II, Bahia	Asteraceae	*Helianthus annuus* L.	Girassol	Seeds	Others	Wounds
Barboza da Silva et al. [21]	2012	Barra II, Bahia	Asteraceae	*Lactuca sativa* L.	Alface	Leaves	Decoction	Insomnia
Barboza da Silva et al. [21]	2012	Barra II, Bahia	Asteraceae	*Matricaria chamomilla* L.	Camomila	Leaves	Decoction	Women’s inflammation; Flu; Urine inflammation
Barboza da Silva et al. [21]	2012	Barra II, Bahia	Asteraceae	*Pluchea sagittalis* (Lam.) Cabrera	Quitoco	Root; Leaves	Decoction; Infusion; Maceration in Alcohol	Urethra pain; Women’s inflammation; Contusion; Leg pain; Menstrual cramps; Stomachache
Barboza da Silva et al. [21]	2012	Barra II, Bahia	Asteraceae	*Sphagneticola trilobata* (L.) Pruski	Calêndula	Whole plant	Maceration; Decoction	Inflammation;
Barboza da Silva et al. [21]	2012	Barra II, Bahia	Asteraceae	*Spondias tuberosa* Arruda	Umbú	Bark	Decoction	Whooping cough
Barboza da Silva et al. [21]	2012	Barra II, Bahia	Asteraceae	*Tagetes patula* L.	Cravo de defunto	Flower	Decoction; Infusion	To avoid dysentery; Stomach pain
Barboza da Silva et al. [21]	2012	Barra II, Bahia	Asteraceae	*Tanacetum parthenium* (L.) Sch. Bip.	Macela/ macela galega	Leaves; Flower	Maceration	Cancer; Women’s inflammation
Barboza da Silva et al. [21]	2012	Barra II, Bahia	Bignoniaceae	*Handroanthus impetiginosus* (Mart. *ex* DC.) Mattos	Pau d’arco roxo	Bark	Others	Evil eye; Back; Contusion forte; Wound healing; Joint pain
Barboza da Silva et al. [21]	2012	Barra II, Bahia	Bignoniaceae	*Tabebuia aurea* (Silva Manso) Benth. and Hook.f. *ex* S.Moore	Caraíba	Bark	Maceration; Infusion	Inflammation; Contusion; Menstrual cramps; Ovaries pain
Barboza da Silva et al. [21]	2012	Barra II, Bahia	Bixaceae	*Bixa orellana* L.	Urucum	Seeds; Leaves	Infusion	Walking aid
Barboza da Silva et al. [21]	2012	Barra II, Bahia	Boraginaceae	*Cordia* sp.	Moleque duro	Leaves	Infusion	Cancer
Barboza da Silva et al. [21]	2012	Barra II, Bahia	Boraginaceae	*Symphytum officinale* L.	Confrei	Leaves	Infusion	Improves memory
Barboza da Silva et al. [21]	2012	Barra II, Bahia	Brassicaceae	*Lepidium ruderale* L.	Morfina	Leaves	Decoction; Infusion	Cough; Phlegm; Flu
Barboza da Silva et al. [21]	2012	Barra II, Bahia	Brassicaceae	*Rorippa nasturtium-aquaticum* (L.) Hayek	Agrião	Leaves	Decoction; Juice	Cholesterol
Barboza da Silva et al. [21]	2012	Barra II, Bahia	Bromeliaceae	*Ananas comosus* (L.) Merril	Abacaxi	Bark	Syrup	Kidney; Back
Barboza da Silva et al. [21]	2012	Barra II, Bahia	Cactaceae	*Melocactus bahiensis* (Britton and Rose) Luetzelb.	Cabeça de frade	Whole plant	Others	Headache
Barboza da Silva et al. [21]	2012	Barra II, Bahia	Cactaceae	*Opuntia ficus-indica* (L.) Mill.	Palma	Others	Decoction; Infusion	Fever; Flu; Cough
Barboza da Silva et al. [21]	2012	Barra II, Bahia	Caricaceae	*Carica papaya* L.	Mamão	Fruit	Maceration; Infusion	Amebiasis; Flu; Phlegm; Pressure; Worms; Diabetes
Barboza da Silva et al. [21]	2012	Barra II, Bahia	Celastraceae	*Monteverdia rigida* (Mart.) Biral	Pau-de-colher	Bark	Maceration in Alcohol; Decoction	Flu
Barboza da Silva et al. [21]	2012	Barra II, Bahia	Commelinaceae	*Murdannia nudiflora* (L.) Brenan	Marianinha	Leaves	Juice	Sight
Barboza da Silva et al. [21]	2012	Barra II, Bahia	Convolvulaceae	*Ipomoea batatas* (L.) Lam.	Batata doce	Leaves	Decoction	Toothache
Barboza da Silva et al. [21]	2012	Barra II, Bahia	Convolvulaceae	*Operculina macrocarpa* (L.) Urb.	Purga de batata	Bark	Decoction	Worms; Hemorrhoids
Barboza da Silva et al. [21]	2012	Barra II, Bahia	Crassulaceae	*Bryophyllum pinnatum* (Lam.) Oken	Folha da costa	Leaves	Decoction	Inflammation; Contusion
Barboza da Silva et al. [21]	2012	Barra II, Bahia	Crassulaceae	*Sedum dendroideum* DC	Bálsamo	Leaves	Juice	Inflammation; Eye pain
Barboza da Silva et al. [21]	2012	Barra II, Bahia	Cucurbitaceae	*Cucurbita* sp.	Abóbora	Seeds	Infusion	Worms
Barboza da Silva et al. [21]	2012	Barra II, Bahia	Cucurbitaceae	*Momordica charantia* L.	São caetano	Leaves	Decoction	Women’s inflammation; Leg pain
Barboza da Silva et al. [21]	2012	Barra II, Bahia	Cucurbitaceae	*Sicyos edulis* Jacq.	Chuchu	Leaves	Decoction	To decrease blood pressure
Barboza da Silva et al. [21]	2012	Barra II, Bahia	Dioscoriaceae	*Dioscorea villosa* L.	Inhame	Root	Decoction	Aphrodisiac
Barboza da Silva et al. [21]	2012	Barra II, Bahia	Erythroxylaceae	*Erythroxylum vacciniifolium* Mart.	Catuaba	Bark	Others	Aphrodisiac
Barboza da Silva et al. [21]	2012	Barra II, Bahia	Euphorbiaceae	*Croton antisyphiliticus* Mart.	Enxerto de passarinho	Bark	Decoction	Toothache
Barboza da Silva et al. [21]	2012	Barra II, Bahia	Euphorbiaceae	*Croton campestris* A. St.-Hil.	Velame	Leaves	Decoction	Stroke; Sight; Mouth wound
Barboza da Silva et al. [21]	2012	Barra II, Bahia	Euphorbiaceae	*Dalechampia* sp.	Urtiga	Root	Decoction	Toothache
Barboza da Silva et al. [21]	2012	Barra II, Bahia	Euphorbiaceae	*Jatropha gossypiifolia* L.	Pinhão roxo	Leaves	Others	Pork allergy
Barboza da Silva et al. [21]	2012	Barra II, Bahia	Euphorbiaceae	*Phyllanthus flaviflorus* (K.Schum. and Lauterb.) Airy Shaw	Quebra pedra	Whole plant	Decoction; Infusion	Kidney pain; Women’s inflammation; Blood pressure
Barboza da Silva et al. [21]	2012	Barra II, Bahia	Euphorbiaceae	*Ricinus communis* L.	Mamona	Others	Others	Helps with childbirth
Barboza da Silva et al. [21]	2012	Barra II, Bahia	Fabaceae	*Abarema cochliacarpos* (Gomes) Barneby and J.W. Grimes	Barbatimão	Bark	Maceration; Maceration in Alcohol; Decoction	Inflammation; Wound healing; Ulcer
Barboza da Silva et al. [21]	2012	Barra II, Bahia	Fabaceae	*Amburana cearensis* (Allem.) A.C. Sm.	Imburana/emburana/umburana	Bark; Leaves; Seeds	Decoction; Infusion	Stomach; Inflammation; Menstrual cramps
Barboza da Silva et al. [21]	2012	Barra II, Bahia	Fabaceae	*Anadenanthera colubrina* (Vell.) Brenan	Angico	Bark	Decoction	Flu; Phlegm
Barboza da Silva et al. [21]	2012	Barra II, Bahia	Fabaceae	*Cajanus cajan* (L.) Huth	Andú branco	Leaves; Flower	Decoction; Infusion	Amebiasis; Stomach pain;
Barboza da Silva et al. [21]	2012	Barra II, Bahia	Fabaceae	*Cenostigma pyramidale* (Tul.) Gagnon and G.P.Lewis	Catinga de porco/pau de rato/manevintura	Leaves	Decoction	Flu; Cough; Sinusitis
Barboza da Silva et al. [21]	2012	Barra II, Bahia	Fabaceae	*Copaifera lucens* Dwyer	Copaiba/ pau de óleo	Others	Others	Stroke; Sight; Throat; Wound healing
Barboza da Silva et al. [21]	2012	Barra II, Bahia	Fabaceae	*Erythrina* sp.	Mulungú	Bark	Decoction	Toothache
Barboza da Silva et al. [21]	2012	Barra II, Bahia	Fabaceae	*Hymenaea stigonocarpa* Mart. *ex* Hayane var. *stigonocarpa*	Jatobá	Bark	Decoction; Infusion; Maceration in Alcohol	Liver, Kidney; Flu
Barboza da Silva et al. [21]	2012	Barra II, Bahia	Fabaceae	*Libidibia ferrea* (Mart. *ex* Tul.) L.P. Queiroz	Pau—ferro	Bark	Maceration in Alcohol; Decoction	Flu
Barboza da Silva et al. [21]	2012	Barra II, Bahia	Fabaceae	*Mimosa* sp.	Jurema	Bark	Decoction	Toothache
Barboza da Silva et al. [21]	2012	Barra II, Bahia	Fabaceae	*Peltophorum* sp.	Farinha seca	Bark	Infusion	Dysentery
Barboza da Silva et al. [21]	2012	Barra II, Bahia	Fabaceae	*Periandra mediterranea* (Vell.) Taub.	Arcaçus	Root	Decoction; In Natura; Maceration in Alcohol	Flu
Barboza da Silva et al. [21]	2012	Barra II, Bahia	Fabaceae	*Senna occidentalis* (L.) Link	Fedegoso	Leaves; Root; Flower	Infusion; Decoction	Flu; Postpartum; Child’s tooth
Barboza da Silva et al. [21]	2012	Barra II, Bahia	Fabaceae	*Tephrosia pupurea* (L.) Pers.	Sena	Leaves	Infusion	Phlegm
Barboza da Silva et al. [21]	2012	Barra II, Bahia	Lamiaceae	*Mentha gentilis* L.	Alevante miúdo	Leaves	Infusion	Blood pressure
Barboza da Silva et al. [21]	2012	Barra II, Bahia	Lamiaceae	*Mentha pulegium* L.	Peijo (poeijo)	Leaves	Decoction; Infusion; Maceration in Alcohol	Bellyache; Fungal infection; Flu; Cough; Postpartum cleansing; Stomach; Throat; Fortification
Barboza da Silva et al. [21]	2012	Barra II, Bahia	Lamiaceae	*Mentha spicata* L.	Hortelã miúdo	Leaves	Juice; Decoction	Amebiasis; Uterus; Stomach; Throat; Fortification; Flu; Worms; To sleep
Barboza da Silva et al. [21]	2012	Barra II, Bahia	Lamiaceae	*Ocimum basilicum* L.	Manjericão	Leaves	Infusion; Decoction	Soothing; Blood pressure; Flu; Cough
Barboza da Silva et al. [21]	2012	Barra II, Bahia	Lamiaceae	*Ocimum carnosum* (Spreng.) Link and Otto *ex* Benth.	Alfavaca	Whole plant	Infusion; Decoction	Flu
Barboza da Silva et al. [21]	2012	Barra II, Bahia	Lamiaceae	*Ocimum* sp.	Basilicão	Leaves	Decoction	Flu
Barboza da Silva et al. [21]	2012	Barra II, Bahia	Lamiaceae	*Plectranthus amboinicus* (Lour.) Spreng.	Hortelã grosso/graúdo	Leaves	Juice; Decoction	Dysentery; Stomachache; Women’s inflammation; Vaginal discharge
Barboza da Silva et al. [21]	2012	Barra II, Bahia	Lamiaceae	*Plectranthus barbatus* Andr.	Boldo/sete dores	Leaves	Infusion; Decoction	Dysentery; Stomach pain; Menstrual cramps
Barboza da Silva et al. [21]	2012	Barra II, Bahia	Lamiaceae	*Plectranthus ornatus* Codd	Boldo de quintal	Leaves	Juice; Infusion; Decoction	Stomach; Kidney; Headache; Fever; Dysentery
Barboza da Silva et al. [21]	2012	Barra II, Bahia	Lamiaceae	*Rosmarinus officinalis* L.	Alecrim de quintal	Whole plant	Decoction; Infusion	Sinusitis; Calmative; Flu, Sore throat
Barboza da Silva et al. [21]	2012	Barra II, Bahia	Lamiaceae	*Salvia officinalis* L.	Salva	Leaves	Infusion; Decoction	Sight; Flu; Headache; Body aches
Barboza da Silva et al. [21]	2012	Barra II, Bahia	Lauraceae	*Cinnamomum verum* J.Presl	Canela	Bark	Decoction; Maceration	Cough, Flu; Uterus; Stomach; Throat; Fortification; Women’s inflammation
Barboza da Silva et al. [21]	2012	Barra II, Bahia	Lauraceae	*Persea americana* Mill.	Abacate	Leaves; Seeds	Decoction; Maceration in Alcohol	Kidney
Barboza da Silva et al. [21]	2012	Barra II, Bahia	Liliaceae	*Nothoscordum* sp.	Alho bravo	Root	Maceration; Maceration in Alcohol; Decoction	Rheumatism
Barboza da Silva et al. [21]	2012	Barra II, Bahia	Lythraceae	*Punica granatum* L.	Romã	Fruit	Decoction	Sore throat
Barboza da Silva et al. [21]	2012	Barra II, Bahia	Malpighiaceae	*Malpighia glabra* L.	Acerola	Leaves; Fruit	Decoction; Infusion; Juice	Bronchitis; Flu
Barboza da Silva et al. [21]	2012	Barra II, Bahia	Malvaceae	*Gossypium herbaceum* L.	Algodão	Fruit; Leaves	Juice; Decoction	Flu; Expectorant; Cough; Pain; Women’s inflammation; Contusion
Barboza da Silva et al. [21]	2012	Barra II, Bahia	Malvaceae	*Luehea grandiflora* Mart	Cedro	Bark	Decoction	Bellyache; Fungal infection; Constipation
Barboza da Silva et al. [21]	2012	Barra II, Bahia	Malvaceae	*Sida cordifolia* L.	Malva—branca	Leaves; Root	Decoction	Blood pressure
Barboza da Silva et al. [21]	2012	Barra II, Bahia	Malvaceae	*Sidastrum micranthum* (A.St.-Hil.) Fryxell	Malva—preta/ malva de sebo	Root	Maceration in Alcohol	Hair loss
Barboza da Silva et al. [21]	2012	Barra II, Bahia	Malvaceae	*Guazuma ulmifolia* Lam.	Mutamba	Bark	Infusion; Decoction	Pain; Flu; Toothache
Barboza da Silva et al. [21]	2012	Barra II, Bahia	Moraceae	*Dorstenia* sp.	Carapuá/carapiá	Root	Decoction	Blood pressure
Barboza da Silva et al. [21]	2012	Barra II, Bahia	Moraceae	*Ficus carica* L.	Figo	Leaves	Decoction	Cough; Blood pressure; Flu
Barboza da Silva et al. [21]	2012	Barra II, Bahia	Musaceae	*Musa sapientum* L.	Banana	Inflorescence	Infusion; Maceration in Alcohol; Decoction	Menstrual cramps; Stroke; Uterus; Stomach; Throat; Fortification
Barboza da Silva et al. [21]	2012	Barra II, Bahia	Myristicaceae	*Myristica fragrans* Houtt.	Noz-moscada/manuscada	Seeds	Infusion; Decoction	Fever; Flu; Sinusitis; Phlegm
Barboza da Silva et al. [21]	2012	Barra II, Bahia	Myrtaceae	*Eucalyptus globulus* Labill.	Eucalipto	Leaves; Seeds	Decoction	Amebiasis; Fever; Flu; Cough
Barboza da Silva et al. [21]	2012	Barra II, Bahia	Myrtaceae	*Eugenia uniflora* L.	Pitanga	Leaves	Decoction	Dysentery
Barboza da Silva et al. [21]	2012	Barra II, Bahia	Myrtaceae	*Plinia peruviana* (Poir.) Govaerts	Jabuticaba	Bark	Decoction	Bellyache; Women’s inflammation
Barboza da Silva et al. [21]	2012	Barra II, Bahia	Myrtaceae	*Psidium guajava* L.	Goiabeira	Bark; Leaves	*In Natura*; Decoction	Headache; Dysentery
Barboza da Silva et al. [21]	2012	Barra II, Bahia	Myrtaceae	*Psidium guineense* Sw.	Araçá	Leaves	Decoction	Cough, Flu, Whooping cough
Barboza da Silva et al. [21]	2012	Barra II, Bahia	Myrtaceae	*Syzygium aromaticum* (L.) Merr. and L.M.Perry	Cravo da índia	Flower	Decoction; Infusion; *In Natura*	Cholesterol; High blood pressure; Ulcer; Diabetes; Heart; Women’s inflammation
Barboza da Silva et al. [21]	2012	Barra II, Bahia	Myrtaceae	*Syzygium cumini* (L.) Skeels	Janelão/jamelão	Leaves; Fruit	Maceration in Alcohol	Postpartum cleansing;
Barboza da Silva et al. [21]	2012	Barra II, Bahia	Nyctaginaceae	*Boerhaavia coccinea* Willd.	Pega pinto	Whole plant	Decoction	To decrease blood pressure
Barboza da Silva et al. [21]	2012	Barra II, Bahia	Orchidaceae	*Vanilla* sp.	Baunilha	Leaves	Infusion	Stroke; Epilepsy
Barboza da Silva et al. [21]	2012	Barra II, Bahia	Papaveraceae	*Argemone mexicana* L.	Carro santo	Leaves	Infusion; Juice; Decoction	To sleep; Blood pressure; Soothing; Hemorrhoids
Barboza da Silva et al. [21]	2012	Barra II, Bahia	Passifloraceae	*Passiflora cincinnata* Mart.	Maracujá do mato	Flower; Fruit; Root	Infusion	Blood pressure
Barboza da Silva et al. [21]	2012	Barra II, Bahia	Passifloraceae	*Passiflora edulis* Sims	Maracujá	Flower; Leaves	Others	Joint pain
Barboza da Silva et al. [21]	2012	Barra II, Bahia	Pedaliaceae	*Sesamum indicum* L.	Gergelin	Seeds	Decoction	Fortification
Barboza da Silva et al. [21]	2012	Barra II, Bahia	Piperaceae	*Piper nigrum* L.	Pimenta do reino	Seeds	Decoction; Infusion; *In Natura*; Maceration in Alcohol	Urine inflammation; Sore throat; Toothache; Women’s inflammation
Barboza da Silva et al. [21]	2012	Barra II, Bahia	Plantaginaceae	*Plantago major* L.	Trançagem	Whole plant	Decoction; Juice	Kidney pain; Back pain; Liver; Toothache
Barboza da Silva et al. [21]	2012	Barra II, Bahia	Poaceae	*Bambusa bambos* (L.) Voss	Bambu	Flower; Fruit	Decoction; Infusion	Soothing; Diabetes; High blood pressure; Flu; Release more urine
Barboza da Silva et al. [21]	2012	Barra II, Bahia	Poaceae	*Cymbopogon citratus* (DC.) Stapf.	Capim-santo	Leaves; Root	Decoction; Infusion; Juice	Headache; Healing
Barboza da Silva et al. [21]	2012	Barra II, Bahia	Poaceae	*Digitaria insularis* (L.) Fedde	Capim açú	Leaves	Decoction	Blood circulation aid
Barboza da Silva et al. [21]	2012	Barra II, Bahia	Poaceae	INDET	Capim lanceta	Aerial part of the plant	Decoction; Infusion	Cough, Flu; Soothing
Barboza da Silva et al. [21]	2012	Barra II, Bahia	Poaceae	INDET	Capim nagô	Aerial part of the plant	Decoction; Infusion	High blood pressure
Barboza da Silva et al. [21]	2012	Barra II, Bahia	Poaceae	*Saccharum officinarum* L.	Cana caiana	Leaves	Others	Toothache
Barboza da Silva et al. [21]	2012	Barra II, Bahia	Poaceae	*Zea mays* L.	Milho	Leaves	Infusion	Heart
Barboza da Silva et al. [21]	2012	Barra II, Bahia	Rosaceae	*Rosa* sp.	Rosa branca ou branca de neve	Flower	Decoction	Stroke
Barboza da Silva et al. [21]	2012	Barra II, Bahia	Rubiaceae	*Coffea* sp.	Café	Seeds	Decoction; maceration in alcohol; In natura	Abortive; Bellyache; Kidney; Liver
Barboza da Silva et al. [21]	2012	Barra II, Bahia	Rubiaceae	*Coutarea hexandra* (Jacq.) K. Schum.	Quina	Bark	Infusion	Pain
Barboza da Silva et al. [21]	2012	Barra II, Bahia	Rubiaceae	*Palicourea coriacea* (Cham.) K. Schum.	Gemedeira	Root	Decoction	Soothing; High blood pressure; Fever; Flu
Barboza da Silva et al. [21]	2012	Barra II, Bahia	Rutaceae	*Citrus aurantium* L.	Laranja	Leaves	Juice; Decoction; Infusion	Cholesterol; High blood pressure; Flu; Skin wound
Barboza da Silva et al. [21]	2012	Barra II, Bahia	Rutaceae	*Citrus x limon* (L.) Osbeck	Limão	Fruit; Leaves	Decoction; Maceration in Alcohol	Phlegm; Flu; Postpartum cleansing; Throat; Fortification
Barboza da Silva et al. [21]	2012	Barra II, Bahia	Rutaceae	*Ruta graveolens* L.	Arruda	Leaves	Maceration; Decoction; Maceration in Alcohol; *in natura*; Infusion	Stomach pain; Dysentery; Vomit; Back pain; Blood pressure
Barboza da Silva et al. [21]	2012	Barra II, Bahia	Smilacaceae	*Smilax hilariana* A.DC.	Jacaré/ catana de jacaré	Root	Decoction	Toothache
Barboza da Silva et al. [21]	2012	Barra II, Bahia	Solanaceae	*Capsicum* sp.	Pimenta	Fruit; Leaves	Decoction	Vaginal discharge; Cystitis
Barboza da Silva et al. [21]	2012	Barra II, Bahia	Solanaceae	*Solanum ambrosiacum* Vell.	Melancia da praia	Whole plant	Decoction	Diarrhea; Worms; Vaginal discharge
Barboza da Silva et al. [21]	2012	Barra II, Bahia	Solanaceae	*Solanum erianthum* D. Don.	Caiçara	Root	Decoction; infusion	To vomit; To stomach pain
Barboza da Silva et al. [21]	2012	Barra II, Bahia	Solanaceae	*Solanum* sp.	Cassutinga	Bark; Leaves	Decoction	Child’s colic
Barboza da Silva et al. [21]	2012	Barra II, Bahia	Sterculiaceae	*Helicteres macropetala* A.St.-Hil.	Rosca	Fruit	Decoction	Stroke
Barboza da Silva et al. [21]	2012	Barra II, Bahia	Verbenaceae	*Aloysia gratíssima* (Gillies and Hook.) Tronc.	Alfazema	Whole plant	Decoction; infusion	Urine inflammation; Flu; Soothing
Barboza da Silva et al. [21]	2012	Barra II, Bahia	Verbenaceae	*Lippia alba* (Mill.) N.E.Br.	Erva cidreira	Leaves	Decoction; Infusion; In Natura	To stomach pain; Dysentery; Barriga fofa; Headache; Pressure; Soothing; Diabetes; Calmative
Barboza da Silva et al. [21]	2012	Barra II, Bahia	Verbenaceae	*Lippia grata* Schauer	Alecrim de vaqueiro	Leaves	Decoction; Infusion	Blood pressure; Flu
Barboza da Silva et al. [21]	2012	Barra II, Bahia	Violaceae	*Pombalia calceolaria* (L.) Paula-Souza	Papaconha	Root	Decoction	Stroke; Amebiasis; Purgative
Barboza da Silva et al. [21]	2012	Barra II, Bahia	Vitaceae	*Vitis aestivalis* Michx.	Uva	Leaves	Decoction	To decrease menstruation
Barboza da Silva et al. [21]	2012	Barra II, Bahia	Xanthorrhoeaceae (Liliaceae)	*Aloe vera* (L.) Burm.f.	Babosa	Leaves	Juice; Decoction	Cancer; Stroke; Worms;
Barboza da Silva et al. [21]	2012	Barra II, Bahia	Zingiberaceae	*Alpinia zerumbet* (Pers.) B.L.Burtt and R.M.Sm.	Alevante vermelho	Root	Infusion	Blood pressure
Beltreschi et al. [22]	2018	Ipiranga, Paraíba	Acanthaceae	*Justicia pectoralis* Jacq.	Xaxaba	Leaves	NR	Flu; Fever; Phlegm; Cough
Beltreschi et al. [22]	2018	Ipiranga, Paraíba	Adoxaceae	*Sambucus australis* Cham. and Schltdl.	Sabugueiro	Leaves; Flower	NR	Flu; Fever; Phlegm; Cough; Hypertension
Beltreschi et al. [22]	2018	Ipiranga, Paraíba	Amaranthaceae	*Alternanthera* aff. *philoxeroides* (Mart.) Griseb.	Acônico	Leaves	NR	Stomachache; Headache; Fever
Beltreschi et al. [22]	2018	Ipiranga, Paraíba	Amaranthaceae	*Dysphania ambrosioides* (L.) Mosyakin and Clemants	Mastruz	Leaves	NR	Anemia; Inflammation; Bronchitis; High cholesterol; Colic; Diabetes; Diarrhea; Phlegm; Gastritis; Flu; Cough; Trauma; Worms
Beltreschi et al. [22]	2018	Ipiranga, Paraíba	Anacardiaceae	*Anacardium occidentale* L.	Cajueiro roxo	Bark	NR	Inflammation; Trauma; Worms; Contusion; Wounds; Stomachache
Beltreschi et al. [22]	2018	Ipiranga, Paraíba	Anacardiaceae	*Schinus terebinthifolia* Raddi	Aroeira	Bark	NR	Inflammation; Sore throat; Trauma; Wounds; Allergies
Beltreschi et al. [22]	2018	Ipiranga, Paraíba	Annonaceae	*Annona muricata* L.	Graviola	Leaves; Fruit	NR	Cancer; Diabetes; Hypertension
Beltreschi et al. [22]	2018	Ipiranga, Paraíba	Apiaceae	*Eryngium foetidum* L.	Coentro maranhão	Leaves	NR	Heart attack
Beltreschi et al. [22]	2018	Ipiranga, Paraíba	Apiaceae	*Pimpinella anisum* L.	Erva-doce	Seeds	NR	Bellyache; Hypertension; Detox; Indigestion
Beltreschi et al. [22]	2018	Ipiranga, Paraíba	Apocynaceae	*Catharanthus roseus* (L.) Don	Boa noite branca	Root	NR	Flu
Beltreschi et al. [22]	2018	Ipiranga, Paraíba	Apocynaceae	*Hancornia speciosa* Gomes	Mangaba	Latex	NR	Gastritis; Ulcer
Beltreschi et al. [22]	2018	Ipiranga, Paraíba	Asteraceae	*Acanthospermum hispidum* DC.	Espinho de cigano	Leaves; Root	NR	Flu; Phlegm; Cough
Beltreschi et al. [22]	2018	Ipiranga, Paraíba	Asparagaceae	*Aloe vera* (L.) Burm. f.	Babosa	Pulp	NR	Flu; Headache; Bellyache
Beltreschi et al. [22]	2018	Ipiranga, Paraíba	Asteraceae	*Bidens* sp.	Camomila	Flower	NR	Sedative; Headache
Beltreschi et al. [22]	2018	Ipiranga, Paraíba	Asteraceae	*Conyza bonariensis* (L.) Cronquist	Rabo de raposa	Leaves	NR	Ringworm
Beltreschi et al. [22]	2018	Ipiranga, Paraíba	Asteraceae	*Gymnanthemum amygdalinum* (Delile) Sch.Bip. *ex* Walp.	Alcachofra	Leaves	NR	High cholesterol; Diabetes; Liver; Kidney pain; Indigestion; Gallbladder pain
Beltreschi et al. [22]	2018	Ipiranga, Paraíba	Asteraceae	*Solidago chilensis* Meyen	Arnica brasileira	Leaves	NR	Contusion
Beltreschi et al. [22]	2018	Ipiranga, Paraíba	Bignoniaceae	*Handroanthus impetiginosus* (Mart. *ex* DC.) Mattos	Pau D’arco roxo	Bark	NR	Inflammation; Anemia; Cancer; Wounds; Trauma
Beltreschi et al. [22]	2018	Ipiranga, Paraíba	Costaceae	*Costus lasius* Loes.	Cana da índia	Leaves	NR	Diabetes; Kidney stone
Beltreschi et al. [22]	2018	Ipiranga, Paraíba	Crassulaceae	*Kalanchoe crenata* (Andrews) Haw.	Saião	Leaves	NR	Gastritis; Flu; Ulcer; Phlegm; Worms
Beltreschi et al. [22]	2018	Ipiranga, Paraíba	Cucurbitaceae	*Cucurbita pepo* L.	Jerimum	Flower	NR	Earache; Hemorrhoids; Worms
Beltreschi et al. [22]	2018	Ipiranga, Paraíba	Cucurbitaceae	*Momordica charantia* L.	Melão São Caetano	Leaves	NR	Sight
Beltreschi et al. [22]	2018	Ipiranga, Paraíba	Euphorbiaceae	*Cnidoscolus urens* (L.) Arthur	Urtiga branca	Root	NR	Inflammation
Beltreschi et al. [22]	2018	Ipiranga, Paraíba	Euphorbiaceae	*Jatropha mollissima* (Pohl) Baill.	Pinhão branco	Latex	NR	Hemorrhage
Beltreschi et al. [22]	2018	Ipiranga, Paraíba	Euphorbiaceae	*Pedilanthus tithymaloides* (L.) Poit.	Beladona	Leaves	NR	Fever; Bellyache; Flu; Headache
Beltreschi et al. [22]	2018	Ipiranga, Paraíba	Euphorbiaceae	*Ricinus communis* L.	Carrapateira	Leaves	NR	Fever; Bellyache; Flu; Headache
Beltreschi et al. [22]	2018	Ipiranga, Paraíba	Fabaceae (Caesalpinoideae)	*Senna occidentalis* (L.) Link	Manjirioba	Leaves	NR	Diabetes; Flu
Beltreschi et al. [22]	2018	Ipiranga, Paraíba	Fabaceae (Cercidoideae)	*Bauhinia monandra* Kurz	Pata de vaca	Leaves	NR	Diabetes
Beltreschi et al. [22]	2018	Ipiranga, Paraíba	Fabaceae (Mimosoideae)	*Stryphnodendron pulcherrimum* (Willd.) Hochr.	Babatenom	Bark	NR	Inflammation; Wounds; Trauma; Contusion
Beltreschi et al. [22]	2018	Ipiranga, Paraíba	Geraniaceae	*Pelargonium graveolens* L’Hér.	Malva-rosa	Whole plant	NR	Fever; Flu; Hemorrhoid; Phlegm
Beltreschi et al. [22]	2018	Ipiranga, Paraíba	Lamiaceae	*Aeollanthus suaveolens* Mart. *ex* Spreng.	Macaça	Leaves	NR	Heart attack; Earache; Hypertension
Beltreschi et al. [22]	2018	Ipiranga, Paraíba	Lamiaceae	*Callicarpa* sp.	Vick	Leaves	NR	Inflammation; Headache; Phlegm; Flu; Fever; Cough
Beltreschi et al. [22]	2018	Ipiranga, Paraíba	Lamiaceae	*Leonotis nepetifolia* (L.) R.Br.	Cordão de São Francisco	Leaves; Flower	NR	Heart attack
Beltreschi et al. [22]	2018	Ipiranga, Paraíba	Lamiaceae	*Mentha* sp	Hortelã miúda	Leaves	NR	Heart attack; Worms; Cough; Amebiasis; Bellyache; Earache; Flu; Hemorrhoid; Sinusitis; Worms; Cough
Beltreschi et al. [22]	2018	Ipiranga, Paraíba	Lamiaceae	*Ocimum basilicum* L	Manjerona	Leaves	NR	Depression
Beltreschi et al. [22]	2018	Ipiranga, Paraíba	Lamiaceae	*Ocimum gratissimum* L.	Louro do mato	Leaves	NR	Diarrhea; Bellyache; Stomachache
Beltreschi et al. [22]	2018	Ipiranga, Paraíba	Lamiaceae	*Plectranthus amboinicus* (Lour.) Spreng.	Hortelã grande	Leaves	NR	Asthma; Bronchitis; Headache; Headache; Sore throat; Phlegm; Flu; Hypertension; Cough; Sinusitis
Beltreschi et al. [22]	2018	Ipiranga, Paraíba	Lamiaceae	*Rosmarinus officinalis* L.	Alecrim	Leaves	NR	Heart attack; Headache; Fever; Hypertension; Thrombosis; Bellyache; Diarrhea
Beltreschi et al. [22]	2018	Ipiranga, Paraíba	Lamiaceae	*Vitex agnus-castus* L.	Liamba	Leaves	NR	Bellyache; Rhinitis
Beltreschi et al. [22]	2018	Ipiranga, Paraíba	Lauraceae	*Cinnamomum verum* J. Presl	Canela	Leaves; Bark	NR	Diarrhea; Stomachache; Vomit
Beltreschi et al. [22]	2018	Ipiranga, Paraíba	Lauraceae	*Persea americana* Mill.	Abacate	Leaves	NR	Bellyache; Kidney pain; Liver; Urinary tract infection; Prostate
Beltreschi et al. [22]	2018	Ipiranga, Paraíba	Lythraceae	*Punica granatum* L.	Romã	Bark; Seeds	NR	Conjunctivitis; Sore throat; Cough
Beltreschi et al. [22]	2018	Ipiranga, Paraíba	Malpighiaceae	*Malpighia glabra* L.	Acerola	Fruit	NR	Flu; Cough
Beltreschi et al. [22]	2018	Ipiranga, Paraíba	Malvaceae	*Guazuma ulmifolia* Lam.	Mutamba	Bark	NR	Dandruff; Diarrhea; Hemorrhoid
Beltreschi et al. [22]	2018	Ipiranga, Paraíba	Meliaceae	*Azadirachta indica* A.Juss.	Nim	Leaves	NR	Ringworm
Beltreschi et al. [22]	2018	Ipiranga, Paraíba	Moraceae	*Morus alba* L.	Amora	Leaves; Fruit	NR	Cramp; High cholesterol; Diabetes; Fever; Gastritis; To lose weight
Beltreschi et al. [22]	2018	Ipiranga, Paraíba	Musaceae	*Musa x paradisiaca* L.	Banana	Latex	NR	Wounds
Beltreschi et al. [22]	2018	Ipiranga, Paraíba	Myrtaceae	*Eugenia uniflora* L.	Pitanga	Leaves; Fruit	NR	Bellyache; Diarrhea; Flu
Beltreschi et al. [22]	2018	Ipiranga, Paraíba	Myrtaceae	*Psidium guajava* L.	Goiaba	Leaves	NR	Bellyache; Diarrhea
Beltreschi et al. [22]	2018	Ipiranga, Paraíba	Myrtaceae	*Psidium guineense* Sw.	Araça	Leaves	NR	Bellyache; Diarrhea
Beltreschi et al. [22]	2018	Ipiranga, Paraíba	Myrtaceae	*Syzygium cumini* (L.) Skeels	Oliveira	Leaves	NR	Diabetes
Beltreschi et al. [22]	2018	Ipiranga, Paraíba	Passifloraceae	*Passiflora edulis* Sims	Maracujá	Leaves; Flower	NR	Sedative
Beltreschi et al. [22]	2018	Ipiranga, Paraíba	Phyllanthaceae	*Phyllanthus niruri* L.	Quebra-pedra	Root	NR	Kidney stone
Beltreschi et al. [22]	2018	Ipiranga, Paraíba	Phytolaccaceae	*Petiveria alliacea* L.	Tipi	Leaves	NR	Wounds; Wounds; Pain
Beltreschi et al. [22]	2018	Ipiranga, Paraíba	Piperaceae	*Piper nigrum* L.	Pimenta do reino	Seeds	NR	Sore throat; Stomachache
Beltreschi et al. [22]	2018	Ipiranga, Paraíba	Plantaginaceae	*Plantago major* L.	Transagem	Leaves	NR	Sore throat
Beltreschi et al. [22]	2018	Ipiranga, Paraíba	Plantaginaceae	*Scoparia dulcis* L.	Vassourinha	Leaves; Flower; Root	NR	Diarrhea; Inflammation; Contusion
Beltreschi et al. [22]	2018	Ipiranga, Paraíba	Poaceae	*Cymbopogon citratus* (DC.) Stapf	Capim santo	Leaves	NR	Anemia; Bellyache; Diarrhea; Diabetes; Stomachache; Flu; Hypertension; Worms; Vomit; Indigestion
Beltreschi et al. [22]	2018	Ipiranga, Paraíba	Rubiaceae	*Borreria verticillata* (L.) G.Mey.	Vassoura de botão	Root; Flower	NR	Bellyache; Diarrhea; Contusion; Constipation; Cough
Beltreschi et al. [22]	2018	Ipiranga, Paraíba	Rubiaceae	*Morinda citrifolia* L.	Noni	Fruit	NR	Heart attack; Cancer; High cholesterol; Diabetes
Beltreschi et al. [22]	2018	Ipiranga, Paraíba	Rubiaceae	*Tocoyena sellowiana* (Cham. and Schltdl.) K.Schum.	Jenipapo bravo	Bark	NR	Contusion; Cough; Inflammation; Wounds; Wounds
Beltreschi et al. [22]	2018	Ipiranga, Paraíba	Rutaceae	*Citrus limon* (L.) Osbeck	Limão	Fruit	NR	Cough; Flu
Beltreschi et al. [22]	2018	Ipiranga, Paraíba	Rutaceae	*Citrus x aurantium* L.	Laranja	Leaves	NR	Sedative; Bellyache; Toothache; Fever
Beltreschi et al. [22]	2018	Ipiranga, Paraíba	Rutaceae	*Ruta graveolens* L.	Arruda	Leaves; Flower	NR	Menstrual cramps; Conjunctivitis; Earache; Fever
Beltreschi et al. [22]	2018	Ipiranga, Paraíba	Solanaceae	*Solanum americanum* Mill.	Erva moura	Leaves; Seeds	NR	Wounds; Contusion
Beltreschi et al. [22]	2018	Ipiranga, Paraíba	Verbanaceae	*Lippia alba* (Mill.) N.E.Br. *ex* P. Wilson	Cidreira	Leaves	NR	Abortive; Anemia; Sedative; Menstrual cramps; Diarrhea; Stomachache; Fever; Flu; Indigestion; Cough
Beltreschi et al. [22]	2018	Ipiranga, Paraíba	Verbanaceae	*Lippia grata* Schauer	Alecrim de tabuleiro	Leaves	NR	Flu
Beltreschi et al. [22]	2018	Ipiranga, Paraíba	Vitaceae	*Cissus verticillata* (L.) Nicolson and C.E.Jarvis	Insulina	Leaves	NR	Diabetes
Beltreschi et al. [22]	2018	Ipiranga, Paraíba	Zingiberaceae	*Alpinia zerumbet* (Pers.) B.L. Burtt and R.M. Sm.	Colônia	Leaves; Flower	NR	Cancer; Acidity; Dandruff; Wounds; Furuncle; Gastritis; Ulcer; Contusion; Hemorrhoids; Worms
Magalhães et al. [23]	2022	Pau D’arco, Alagoas	Acanthaceae	*Ruellia* sp.	Solda-osso	Whole plant	NR	Bronchitis; Phlegm; Headache; Fever; Flu; Sinusitis; Cough
Magalhães et al. [23]	2022	Pau D’arco, Alagoas	Adoxaceae	*Sambucus nigra* L.	Sabugueira	Flower	Infusion	Broken bone
Magalhães et al. [23]	2022	Pau D’arco, Alagoas	Amaranthaceae	*Alternanthera tenella* Colla	Meracilina	Leaves	Boiled	Fever; Sore throat
Magalhães et al. [23]	2022	Pau D’arco, Alagoas	Amaranthaceae	*Dysphania ambrosioides* (L.) Mosyakin and Clemants	Mentruz	Leaves; Stem	NR	Pain; Inflammation
Magalhães et al. [23]	2022	Pau D’arco, Alagoas	Apiaceae	*Apium graveolens* L.	Endro	Leaves; Bark	NR	Worms; Cough
Magalhães et al. [23]	2022	Pau D’arco, Alagoas	Asparagaceae	*Asparagus densiflorus* (Kunth) Jessop	Alfinete/agulha	Leaves; Stem	NR	Anemia; Inflammation
Magalhães et al. [23]	2022	Pau D’arco, Alagoas	Asparagaceae	*Dracaena trifasciata* (Prain) Mabb.	Espada-de-são-jorge	Leaves	Boiled	To avoid stroke
Magalhães et al. [23]	2022	Pau D’arco, Alagoas	Asteraceae	*Solidago chilensis* Meyen	Arnica	Leaves; Stem; Flower	NR	Energetic
Magalhães et al. [23]	2022	Pau D’arco, Alagoas	Cactaceae	*Pereskia grandifolia* Haw.	Orai-por- nove	Leaves; Flower; Fruit	NR	Back pain; Pain; Muscle torsion
Magalhães et al. [23]	2022	Pau D’arco, Alagoas	Euphorbiaceae	*Croton heliotropiifolius* Kunth	Velande	Leaves	NR	Pain
Magalhães et al. [23]	2022	Pau D’arco, Alagoas	Euphorbiaceae	*Jatropha gossypiifolia* L.	Pião-roxo	Leaves; Stem	NR	Diabetes; Inflammation
Magalhães et al. [23]	2022	Pau D’arco, Alagoas	Euphorbiaceae	*Jatropha multifida* L.	Metiolate	Leaves	NR	Wound healing; Muscle relaxant; Kills insects
Magalhães et al. [23]	2022	Pau D’arco, Alagoas	Fabaceae	*Cajanus cajan* (L.) Huth	Feijão-andu	Leaves; Seeds	NR	Wound healing
Magalhães et al. [23]	2022	Pau D’arco, Alagoas	Fabaceae	*Senna macranthera* (DC.) H.S.Irwin and Barneby	Fedegoso	Seeds	NR	Headache; Labyrinthitis; Depression; High blood pressure
Magalhães et al. [23]	2022	Pau D’arco, Alagoas	Lamiaceae	*Melissa officinalis* L.	Erva-cidreira	Leaves	NR	Labyrinthitis; High blood pressure
Magalhães et al. [23]	2022	Pau D’arco, Alagoas	Lamiaceae	*Mentha arvensis* L.	Vick	Leaves	NR	Cramp; Depression
Magalhães et al. [23]	2022	Pau D’arco, Alagoas	Lamiaceae	*Mentha suaveolens* Ehrh	Hortelã-da-folha-miúda	Leaves	NR	Cramp; Inflammation; Stomachache
Magalhães et al. [23]	2022	Pau D’arco, Alagoas	Lamiaceae	*Mesosphaerum pectinatum* (L.) Kuntze	Favaca-pequena	Leaves; Stem	NR	Cramp; Flu; Ingestion; Inflammation
Magalhães et al. [23]	2022	Pau D’arco, Alagoas	Lamiaceae	*Ocimum basilicum* L.	Sambacaitá	Leaves	NR	Cramp
Magalhães et al. [23]	2022	Pau D’arco, Alagoas	Lamiaceae	*Ocimum* sp.	Favaca-de-vaqueiro	Leaves; Root	NR	Inflammation
Magalhães et al. [23]	2022	Pau D’arco, Alagoas	Lamiaceae	*Salvia rosmarinoides* A.St.-Hil.	Alecrim	Leaves; Stem	NR	Cramp; Depression; Ingestion
Magalhães et al. [23]	2022	Pau D’arco, Alagoas	Lamiaceae	*Vitex* sp.	Jurema-de-cabloco	Leaves; Seeds	Infusion	To prevent heart attack; Hoarseness
Magalhães et al. [23]	2022	Pau D’arco, Alagoas	Malpighiaceae	*Byrsonima stipulacea* A.Juss	Murici	Leaves; Fruit	NR	Headache; Infertility; To control menstrual period; To relieve menopausal heat; To reduce male libido
Magalhães et al. [23]	2022	Pau D’arco, Alagoas	Phyllantaceae	*Phyllanthus niruri* L.	Quebra-pedra	Leaves; Root	NR	Fever; Inflammation; Cancer
Magalhães et al. [23]	2022	Pau D’arco, Alagoas	Phytolaccaceae	*Petiveria alliacea* L.	Tipi	Leaves	NR	Kidney stone; Diabetes
Magalhães et al. [23]	2022	Pau D’arco, Alagoas	Plantaginaceae	*Plantago major* L.	Transagem	Leaves	NR	Energetic; Joint pain
Magalhães et al. [23]	2022	Pau D’arco, Alagoas	Polygalaceae	*Polygala microphylla* L.	Zezinho	Leaves; Stem	NR	Urinary tract infection; Ovarian infection; Cancer
Magalhães et al. [23]	2022	Pau D’arco, Alagoas	Pteridaceae	*Adiantium capillus-veneri* L.	Avenca	Leaves; Stem	Infusion	Swelling; Postpartum
Magalhães et al. [23]	2022	Pau D’arco, Alagoas	Rubiaceae	*Morinda citrifolia* L.	Noni	Fruit	NR	Cancer; Diabetes; Hypertension
Magalhães et al. [23]	2022	Pau D’arco, Alagoas	Verbenaceae	*Lippia alba* (Mill.) N.E.Br. *ex* Britton and P.Wilson	Beladona	Leaves; Stem	NR	Pneumonia; Diabetes; Kidney stone
Oliveira et al. [24]	2015	Oriximiná, Pará	Anacardiaceae	*Mangifera indica* L.	manga- grande, mangueira	Bark	NR	Depression; Diabetes
Oliveira et al. [24]	2015	Oriximiná, Pará	Apocynaceae	*Aspidosperma excelsum* Benth.	Carapanaúba	Bark	NR	Malaria
Oliveira et al. [24]	2015	Oriximiná, Pará	Apocynaceae	*Geissospermum argenteum* Woodson	Quinarana	Bark	NR	Malaria; Fever; Liver; Body aches, Migraine
Oliveira et al. [24]	2015	Oriximiná, Pará	Apocynaceae	*Himatanthus articulates* (Vahl) Woodson	Sucuuba	Latex	NR	Liver; Malaria; Hepatitis
Oliveira et al. [24]	2015	Oriximiná, Pará	Arecaceae	*Euterpe ptrvstoria* Mart.	Açaí	Root	NR	Malaria; Fortification
Oliveira et al. [24]	2015	Oriximiná, Pará	Asteraceae	*Artemisia vulgaris* L.	Anador	Aerial part of the plant	NR	Anemia; Hepatitis; Jaundice; Liver; Fatigue; Malaria
Oliveira et al. [24]	2015	Oriximiná, Pará	Asteraceae	*Bidens bipinnata* L.	Picão/carrapicho	Root	NR	Headache; Malaria; Fever; Body aches
Oliveira et al. [24]	2015	Oriximiná, Pará	Asteraceae	*Blainvillea acmella* (L.) Philipson	Jambu, jambuí	Aerial part of the plant	NR	Malaria, Liver
Oliveira et al. [24]	2015	Oriximiná, Pará	Asteraceae	*Gymnanthemum amygdalinum* (Delile) Sch.Bip. *ex* Walp	Figatil	Leaves	NR	Liver; Malaria; Migraine
Oliveira et al. [24]	2015	Oriximiná, Pará	Caricaceae	*Carica papaya* L.	Mamão- macho	Leaves	NR	Liver; Malaria; Restlessness; Nausea; Fever; Hepatitis
Oliveira et al. [24]	2015	Oriximiná, Pará	Convolvulaceae	*Operculina hamiltonii* (G.Don) D.F. Austin and Staples	Batatão, batata-depurga	Tuber	NR	Malaria; Liver; Nausea; Anemia, Laxative; Vomit; Spleen cleansing, Restlessness
Oliveira et al. [24]	2015	Oriximiná, Pará	Curcubitaceae	*Citrullus lanatus* (Thunb.) Matsum. and Nakai	Melancia	Seeds	NR	Laxative; Clearance; Malaria; Hepatitis
Oliveira et al. [24]	2015	Oriximiná, Pará	Curcubitaceae	*Luffa operculata* (L.) Cogn	Cabacinha	Fruit	NR	Malaria
Oliveira et al. [24]	2015	Oriximiná, Pará	Euphorbiaceae	*Croton cajucara* Benth.	Sacaca	Bark	NR	Emetic; Malaria
Oliveira et al. [24]	2015	Oriximiná, Pará	Euphorbiaceae	*Croton sacaquinha* Croizat	Sacaquinha, piaçoca	Bark	NR	Liver, Malaria, Hepatitis
Oliveira et al. [24]	2015	Oriximiná, Pará	Euphorbiaceae	*Jatropha curcas* L.	Peão-branco	Seeds	NR	Liver; Malaria; Migraine
Oliveira et al. [24]	2015	Oriximiná, Pará	Euphorbiaceae	*Meororis stipulate* Raf.	Quebra-pedra	Whole plant	NR	Laxative; Malaria; Loss of appetite
Oliveira et al. [24]	2015	Oriximiná, Pará	Fabaceae	*Dalbergia riedelii* (Benth.) Sandwith	Verônica	Bark	NR	Malaria; Jaundice
Oliveira et al. [24]	2015	Oriximiná, Pará	Fabaceae	*Machaerium ferox* (Benth.) Ducke	Saratudo	Trunk	NR	Anemia; Malaria
Oliveira et al. [24]	2015	Oriximiná, Pará	Fabaceae	*Senna occidentalis* Link.	Paramagioba	Root	NR	Malaria; Jaundice
Oliveira et al. [24]	2015	Oriximiná, Pará	Humiriaceae	*Endopleura uchi* (Huber) Cuatrec	Uxi-liso	Bark	NR	Malaria; Anemia
Oliveira et al. [24]	2015	Oriximiná, Pará	Lamiaceae	*Plectranthus barbatus* Andr.	Melhoral, boldo	Leaves	NR	Malaria; Liver; Anemia
Oliveira et al. [24]	2015	Oriximiná, Pará	Lauraceae	*Cinnamomum verum* J.Presl	Canela	Leaves	NR	Liver; Hangover; Malaria; Migraine; Anemia
Oliveira et al. [24]	2015	Oriximiná, Pará	Lecythidaceae	*Bertholletia excelsa* Bonpl.	Castanheira	Fruit	NR	Fatigue; Headache; Migraine; Liver; Malaria
Oliveira et al. [24]	2015	Oriximiná, Pará	Malvaceae	*Quararibea guianensis* Aubl.	Inajarana	Bark	NR	Malaria; Jaundice
Oliveira et al. [24]	2015	Oriximiná, Pará	Meliaceae	*Carapa guianensis* Aubl.	Andiroba	Seeds	NR	Anemia; Hepatitis; Malaria; Liver
Oliveira et al. [24]	2015	Oriximiná, Pará	Meliaceae	*Cedrela odorata* L.	Cedro	Bark	NR	Malaria
Oliveira et al. [24]	2015	Oriximiná, Pará	Moraceae	*Parahancornia fasciculata* (Poir.) Benoist	Amapá- amargo	Latex	NR	Malaria
Oliveira et al. [24]	2015	Oriximiná, Pará	Pedaliaceae	*Sesamum indicum* L.	Gergelim	Seeds	NR	To prevent malaria; Improves blood circulation
Oliveira et al. [24]	2015	Oriximiná, Pará	Rhamnaceae	*Ampelozizyphus amazonicus* Ducke	Saracuramirá	Bark	NR	Malaria
Oliveira et al. [24]	2015	Oriximiná, Pará	Rubiaceae	*Uncaria guianensis* (Aubl.) J.F.Gmel.	Unha-de-gato	Bark	NR	Malaria; Liver; Clearance; Anemia; Loss of appetite
Oliveira et al. [24]	2015	Oriximiná, Pará	Rutaceae	*Citrus x aurantium* L.	Laranja-daterra, laranjeira	Fruit	NR	Anemia; Malaria
Oliveira et al. [24]	2015	Oriximiná, Pará	Rutaceae	*Ruta graveolens* L.	Arruda	Aerial part of the plant	NR	Liver; Malaria; Anemia; Fatigue; Headache; Migraine
Oliveira et al. [24]	2015	Oriximiná, Pará	Simaroubaceae	*Homololepis cedron* (Planch.) Devecchi and Pirani	Pau-paratudo	Bark	NR	Fever; Malaria; Headache; Body aches; Fatigue; To prevent diseases
Oliveira et al. [24]	2015	Oriximiná, Pará	Solanaceae	*Physalis angulata* L.	Gamapu, camapu	Root	NR	Malaria
Oliveira et al. [24]	2014	Oriximiná, Pará	Verbenaceae	*Lippia origanoides* Kunth	Salva-de-marajó	Leaves; Aerial part of the plant	NR	Liver; Anemia; Hepatitis; Malaria
Rodrigues [25]	2007	Sesmaria Mata-Cavalos, Mato Grosso	Arecaceae	*Syagrus petraea* (Mart.) Becc	Açaí-bravo	Fruit	NR	Menstrual cramps, Stomachache; Baby colic; Postpartum.
Rodrigues [25]	2007	Sesmaria Mata-Cavalos, Mato Grosso	Lamiaceae	*Hyptidendron canum* (Pohl *ex* Benth.) Harley	Hortela-da-várzea	Leaves	*In Natura*	Contraceptive
Rodrigues [25]	2007	Sesmaria Mata-Cavalos, Mato Grosso	Loganiaceae	*Strychnos pseudoquina* A. St.-Hil	Quina	Leaves; Bark	Decoction	Abortive
Rodrigues [25]	2007	Sesmaria Mata-Cavalos, Mato Grosso	Oxalidaceae	*Oxalis physocalyx* Zucc. *ex* Progel	Azedinha	Whole plant	Decoction	Abortive
Rodrigues and Carlini [26]	2004	Sesmaria Mata-Cavalos, Mato Grosso	Asteraceae	*Conyza bonariensis* (L.) Cronquist	NI	NI	Juice	Abortive
Rodrigues and Carlini [26]	2006	Sesmaria Mata-Cavalos, Mato Grosso	Bignoniaceae	*Cybistax antisyphilitica* (Mart.) Mart	NI	NI	NR	Mental alteration
Rodrigues and Carlini [26]	2004	Sesmaria Mata-Cavalos, Mato Grosso	Fabaceae	*Senna occidentalis* (L.) Link	NI	NI	NR	Headache
Rodrigues and Carlini [26]	2004	Sesmaria Mata-Cavalos, Mato Grosso	Flacourtiaceae	*Casearia sylvestris* Sw.	NI	NI	NR	Mental alteration
Rodrigues and Carlini [26]	2004	Sesmaria Mata-Cavalos, Mato Grosso	Lythraceae	*Lafoensia pacari* A. St.-Hil.	NI	NI	NR	Mental alteration
Rodrigues and Carlini [26]	2006	Sesmaria Mata-Cavalos, Mato Grosso	Malpighiaceae	*Heteropterys tomentosa* A.Juss.	Nó-de-Cachorro	NI	NR	Mental alteration
Rodrigues and Carlini [26]	2004	Sesmaria Mata-Cavalos, Mato Grosso	Malvaceae (Sterculiaceae)	*Guazuma ulmifolia* Lam.	NI	NI	NR	Rejuvenating
Rodrigues and Carlini [26]	2004	Sesmaria Mata-Cavalos, Mato Grosso	Moraceae	*Brosimum gaudichaudii* Trécul.	Algodaozinho	NI	NR	Mental alteration
Rodrigues and Carlini [26]	2006	Sesmaria Mata-Cavalos, Mato Grosso	Phytolaccaceae	*Petiveria alliacea* L.	NI	NI	Decoction	Mental alteration
Rodrigues and Carlini [26]	2006	Sesmaria Mata-Cavalos, Mato Grosso	Poaceae	*Cymbopogon citratus* (DC.) Stapf	NI	NI	NR	Mental alteration
Rodrigues and Carlini [26]	2004	Sesmaria Mata-Cavalos, Mato Grosso	Rutaceae	*Citrus sinensis* (L.) Osbeck	NI	NI	NR	Soothing; To decrease blood pressure
Rodrigues et al. [27]	2008	Sesmaria Mata-Cavalos, Mato Grosso	Amaryllidaceae	*Allium sativum* L.	Alho	Bulb	NR	Mental alteration
Rodrigues et al. [27]	2008	Sesmaria Mata-Cavalos, Mato Grosso	Bignoniaceae	*Anemopaegma arvense* (Vell.) Stellfeld *ex* de Souza	Alecrim-do-norte	Leaves	NR	To improve learning; Nervous breakdown
Rodrigues et al. [27]	2008	Sesmaria Mata-Cavalos, Mato Grosso	Lamiaceae	*Hyptidendron canum* (Pohl *ex* Benth.) Harley	hortelã-da-várzea	Leaves	NR	To improve learning; Nervous breakdown
Rodrigues et al. [27]	2008	Sesmaria Mata-Cavalos, Mato Grosso	Moraceae	*Dorstenia asaroides* Gardner	Caiá-piá	Rhizome	NR	To improve learning; Nervous breakdown
Rodrigues et al. [27]	2008	Sesmaria Mata-Cavalos, Mato Grosso	Myrtaceae	*Eucalyptus globulus* Labill	Eucalipto	Leaves	NR	To improve learning; Nervous breakdown
Rodrigues et al. [27]	2008	Sesmaria Mata-Cavalos, Mato Grosso	Myrtaceae	*Syzygium aromaticum* (L.) Merr. and L.M.Perry	Cravo-da-Índia	Flower	NR	To improve learning; Nervous breakdown
Rodrigues et al. [27]	2008	Sesmaria Mata-Cavalos, Mato Grosso	Phytolaccaceae	*Petiveria alliacea* L.	Guiné	Leaves	NR	To improve learning; Nervous breakdown
Rodrigues et al. [27]	2008	Sesmaria Mata-Cavalos, Mato Grosso	Rutaceae	*Ruta graveolens* L.	Arruda	Leaves	NR	To improve learning; Nervous breakdown
Rodrigues et al. [27]	2008	Sesmaria Mata-Cavalos, Mato Grosso	Siparunaceae	*Siparuna guianensis* Aubl.	Negramina	Leaves	NR	To improve learning; Nervous breakdown
Yazbek et al. [28]	2019	Quilombo da Fazenda, São Paulo	Acanthaceae	*Justicia pectoralis* Jacq.	Doril	Whole plant	NR	To improve learning; Nervous breakdown
Yazbek et al. [28].	2019	Quilombo da Fazenda, São Paulo	Adoxaceae	*Sambucus* cf. *canadensis* L.	Sabugueiro	Leaves; Flower	Decoction	Flu; Headache
Yazbek et al. [28]	2019	Quilombo da Fazenda, São Paulo	Alismataceae	*Echinodorus grandiflorus* (Cham. and Schltr.) Micheli	Chapéu-de-couro	Leaves	Decoction/Syrup	Fever; Measles
Yazbek et al. [28]	2019	Quilombo da Fazenda, São Paulo	Amaranthaceae	*Alternanthera brasiliana* (L.) Kuntze	Terramicina	Leaves	Decoction	Kidney stone; Diabetes
Yazbek et al. [28]	2019	Quilombo da Fazenda, São Paulo	Amaranthaceae	*Dysphania ambrosioides* (L.) Mosyakin and Clemants	Erva-de-Santa Maria	Leaves	Decoction	Flu; Headache; Urinary tract infection
Yazbek et al. [28]	2019	Quilombo da Fazenda, São Paulo	Amaranthaceae	*Pfaffia glomerata* (Spreng.) Pedersen	Novalgina	Leaves	Infusion	Wounds; Bone trauma; To prevent worms
Yazbek et al. [28]	2019	Quilombo da Fazenda, São Paulo	Anacardiaceae	*Anacardium occidentale* L.	Cajueiro	Bark	Decoction	Flu; Headache; Fever
Yazbek et al. [28]	2019	Quilombo da Fazenda, São Paulo	Annonaceae	*Annona muricata* L.	Graviola	Leaves	Decoction	Swelling; Hemorrhoid; Bone trauma
Yazbek et al. [28]	2019	Quilombo da Fazenda, São Paulo	Apiaceae	*Eryngium foetidum* L.	Coentro-natural	Whole plant	Decoction	Diabetes
Yazbek et al. [28]	2019	Quilombo da Fazenda, São Paulo	Apiaceae	*Foeniculum vulgare* Mill.	Erva-doce	Whole plant	Decoction	Snakebite
Yazbek et al. [28]	2019	Quilombo da Fazenda, São Paulo	Apocynaceae	*Tabernaemontana laeta* Mart.	Guaraná	Exudate	Decoction	Cold; Soothing
Yazbek et al. [28]	2019	Quilombo da Fazenda, São Paulo	Araceae	*Colocasia esculenta* (L.) Schott	Inhame	Root	*In Natura*	Myiasis
Yazbek et al. [28]	2019	Quilombo da Fazenda, São Paulo	Araceae	*Philodendron martianum* Engl.	Banana-do-mato	Exudate	Cooked	Anemia
Yazbek et al. [28]	2019	Quilombo da Fazenda, São Paulo	Asparagaceae	*Furcraea foetida* (L.) Haw	Pita	Leaves	*In Natura*	Dandruff
Yazbek et al. [28]	2019	Quilombo da Fazenda, São Paulo	Asteraceae	*Achillea millefolium* L.	Camomila	Whole plant	Maceration	Scabies
Yazbek et al. [28]	2019	Quilombo da Fazenda, São Paulo	Asteraceae	*Acmella ciliata* (Kunth) Cass.	Anestesia	Bark	Decoction	Expectorant; Helminthiasis
Yazbek et al. [28]	2019	Quilombo da Fazenda, São Paulo	Asteraceae	*Ageratum conyzoides* L.	Erva-de-São-João	Leaves	Decoction	Headache
Yazbek et al. [28]	2019	Quilombo da Fazenda, São Paulo	Asteraceae	*Baccharis* sp.	Carqueja	Leaves	Maceration	Bruises; Cold; Bone trauma; Menstrual regulation
Yazbek et al. [28]	2019	Quilombo da Fazenda, São Paulo	Asteraceae	*Bidens pilosa* L.	Picão	Whole plant	Maceration	Diarrhea
Yazbek et al. [28]	2019	Quilombo da Fazenda, São Paulo	Asteraceae	*Conyza* cf. *canadensis* (L.) Cronquist	Taporava	Leaves	Decoction	Anemia; Hepatitis
Yazbek et al. [28]	2019	Quilombo da Fazenda, São Paulo	Asteraceae	*Emilia sonchifolia* (L.) DC	Serralha	Leaves	Heated	Antifungal
Yazbek et al. [28]	2019	Quilombo da Fazenda, São Paulo	Asteraceae	*Erechtites valerianifolius* (Wolf) DC	Gondó	Whole plant	Decoction	Gastritis
Yazbek et al. [28]	2019	Quilombo da Fazenda, São Paulo	Asteraceae	*Gamochaeta pensylvanica* (Willd.) Cabrera	Macelinha	Whole plant	*In natura*	Clearance; Anemia
Yazbek et al. [28]	2019	Quilombo da Fazenda, São Paulo	Asteraceae	*Gymnanthemum amygdalinum* (Delile) Sch.Bip. *ex* Walp.	Boldo-sem-pêlo	Leaves	Decoction	Constipation
Yazbek et al. [28]	2019	Quilombo da Fazenda, São Paulo	Asteraceae	*Mikania laevigata* Sch.Bip. *ex* Bake	Guaco	Leaves	Decoction	Digestive
Yazbek et al. [28]	2019	Quilombo da Fazenda, São Paulo	Asteraceae	*Montanoa bipinnatifida* (Kunth) K.Koch	Flor-de-maio	Leaves	Decoction	Cough; Sore throat
Yazbek et al. [28]	2019	Quilombo da Fazenda, São Paulo	Asteraceae	*Vernonanthura beyrichii* (Less.) H.Rob.	Cambará-preto/Cambará-roxo	Leaves	Decoction	Ulcer
Yazbek et al. [28]	2019	Quilombo da Fazenda, São Paulo	Bignoniaceae	*Handroanthus impetiginosus* (Mart. *ex* DC.) Mattos	Ipê-roxo	Bark	Decoction	Bruises; Pneumonia; Bone trauma
Yazbek et al. [28]	2019	Quilombo da Fazenda, São Paulo	Bignoniaceae	*Jacaranda puberula* Cham.	Carobinha	Leaves	Decoction	Clearance; Anemia
Yazbek et al. [28]	2019	Quilombo da Fazenda, São Paulo	Bignoniaceae	*Varronia curassavica* Jacq.	Erva-baleeira	Leaves	Decoction	Coagulant
Yazbek et al. [28]	2019	Quilombo da Fazenda, São Paulo	Bromeliaceae	*Bromelia antiacantha* Bertol.	Picova-amarelo	Fruit	Maceration	Bruises; Bone trauma; Back pain; Myoma
Yazbek et al. [28]	2019	Quilombo da Fazenda, São Paulo	Caricaceae	*Carica papaya* L.	Mamão	Flower	Decoction	Flu; Bronchitis
Yazbek et al. [28]	2019	Quilombo da Fazenda, São Paulo	Celastraceae	*Monteverdia ilicifolia* (Mart. *ex* Reissek) Biral	Espinheira-Santa	Leaves	*In natura*	Cough
Yazbek et al. [28]	2019	Quilombo da Fazenda, São Paulo	Clusiaceae	*Garcinia gardneriana* (Planch. and Triana) Zappi	Bacupari	Bark	Decoction	Stomachache
Yazbek et al. [28]	2019	Quilombo da Fazenda, São Paulo	Convolvulaceae	*Cuscuta obtusiflora* Kunth	Cipó-chumbo	Whole plant	Decoction	Gastritis
Yazbek et al. [28]	2019	Quilombo da Fazenda, São Paulo	Convolvulaceae	*Ipomoea batatas* (L.) Lam	Batata	Leaves	Decoction	Scabies
Yazbek et al. [28]	2019	Quilombo da Fazenda, São Paulo	Costaceae	*Costus arabicus* L.	Caninha-do-brejo	Whole plant	Heated	Toothache
Yazbek et al. [28]	2019	Quilombo da Fazenda, São Paulo	Crassulaceae	*Kalanchoe pinnata* (Lam.) Pers	Saião-roxo	Leaves	Decoction	Urinary tract infection
Yazbek et al. [28]	2019	Quilombo da Fazenda, São Paulo	Crassulaceae	*Kalanchoe pinnata* (Lam.) Pers	Saião-branco	Leaves	Maceration	Ulcer
Yazbek et al. [28]	2019	Quilombo da Fazenda, São Paulo	Crassulaceae	*Sedum* cf. *dendroideum* Moc. and Sessé ex DC.	Bálsamo	Leaves	Heated	Antifungal
Yazbek et al. [28]	2019	Quilombo da Fazenda, São Paulo	Cucurbitaceae	*Cucurbita* cf. *maxima* Duchesne	Abóbora	Flower; Seeds	Raw, unprocessed	Digestion
Yazbek et al. [28]	2019	Quilombo da Fazenda, São Paulo	Cucurbitaceae	*Momordica charantia* L.	Malãozinho-do-mato	Leaves	Heated	Earache
Yazbek et al. [28]	2019	Quilombo da Fazenda, São Paulo	Dilleniaceae	*Davilla rugosa* Poir	Cipó-caboclo	Exudate	Decoction	Ulcer
Yazbek et al. [28]	2019	Quilombo da Fazenda, São Paulo	Euphorbiaceae	*Euphorbia thymifolia* L.	Quebra-pedra-roxo	Whole plant	Maceration	Scabies
Yazbek et al. [28]	2019	Quilombo da Fazenda, São Paulo	Euphorbiaceae	*Manihot esculenta* Crantz	Mandioca-doce	Leaves	*In natura*	Cataract
Yazbek et al. [28]	2019	Quilombo da Fazenda, São Paulo	Fabaceae	*Bauhinia forficata* Link	Pata-de-vaca	Leaves	Decoction	To prevent kidney stone
Yazbek et al. [28]	2019	Quilombo da Fazenda, São Paulo	Fabaceae	*Hymenaea altissima* Ducke	Jatobá/Jataí	Bark; Exudate	Cooked	Fortification
Yazbek et al. [28]	2019	Quilombo da Fazenda, São Paulo	Fabaceae	*Mimosa pudica* L.	Dormideira	Leaves	Decoction	Diabetes
Yazbek et al. [28]	2019	Quilombo da Fazenda, São Paulo	Fabaceae	*Swartzia oblata* R.S.Cowan.	Barbatimão	Bark; Leaves	Decoction	Coagulant; Anemia; Digestion; Diabetes; Fortification
Yazbek et al. [28]	2019	Quilombo da Fazenda, São Paulo	Hypoxidaceae	*Hypoxis decumbens* L.	Cebolinha-do-mato	Bulb	Infusion	Sore throat; To sleep better
Yazbek et al. [28]	2019	Quilombo da Fazenda, São Paulo	Lamiaceae	*Mentha pulegium* L.	Poejo	Leaves	Decoction	Coagulant; Anti-inflammatory; Back pain; Scabies
Yazbek et al. [28]	2019	Quilombo da Fazenda, São Paulo	Lamiaceae	*Mentha* sp.	Hortelã-de-bicha	Leaves	Decoction	Diabetes
Yazbek et al. [28]	2019	Quilombo da Fazenda, São Paulo	Lamiaceae	*Ocimum gratissimum* L.	Favacão	Leaves	Decoction	Expectorant; Sore throat
Yazbek et al. [28]	2019	Quilombo da Fazenda, São Paulo	Lamiaceae	*Plectranthus amboinicus* (Lour.) Spreng	Hortelã-castelo/Hortelã-de-carne	Leaves	Decoction	Flu; Soothing; Helminthiasis
Yazbek et al. [28]	2019	Quilombo da Fazenda, São Paulo	Lamiaceae	*Plectranthus barbatus* Andrews	boldo-com-pelo	Leaves	Syrup	Cough
Yazbek et al. [28]	2019	Quilombo da Fazenda, São Paulo	Lauraceae	*Cryptocarya mandioccana* Meisn.	Noz-moscada	Seeds	Decoction	Flu
Yazbek et al. [28]	2019	Quilombo da Fazenda, São Paulo	Lauraceae	*Cryptocarya saligna* Mez	Canela-sassafraize	Bark	Decoction	Hangover
Yazbek et al. [28]	2019	Quilombo da Fazenda, São Paulo	Lauraceae	*Persea americana* Mill.	Abacate-roxo	Leaves	Decoction; Maceration	Bruises; Ulcer; Bronchitis; Bone trauma
Yazbek et al. [28]	2019	Quilombo da Fazenda, São Paulo	Loranthaceae	*Struthanthus marginatus* (Desr.) G.Don	Erva-de-passarinho	Leaves	Maceration	Purification; Measles
Yazbek et al. [28]	2019	Quilombo da Fazenda, São Paulo	Lythraceae	*Cuphea carthagenensis* (Jacq.) J.F.Macbr.	Sete-sangria	Whole plant	Decoction	Kidney stone
Yazbek et al. [28]	2019	Quilombo da Fazenda, São Paulo	Lythraceae	*Psidium cattleianum* Sabine	Araçá	Leaves	Maceration	Coagulant; Sore throat; Bone trauma
Yazbek et al. [28]	2019	Quilombo da Fazenda, São Paulo	Malvaceae	*Sida planicaulis* Cav.	Vassoura-guanxuma	Leaves	Maceration	Digestion
Yazbek et al. [28]	2019	Quilombo da Fazenda, São Paulo	Meliaceae	*Cedrela fissilis* Vell.	Cedro-rosa	Bark	Decoction	Antihypertensive; Hepatitis; To prevent kidney stone
Yazbek et al. [28]	2019	Quilombo da Fazenda, São Paulo	Musaceae	*Musa x paradisiaca* L.	Banana	Exudate	Decoction	Diarrhea
Yazbek et al. [28]	2019	Quilombo da Fazenda, São Paulo	Myrtaceae	*Syzygium jambos* (L.) Alston	Jambo/jambolão	Leaves	Heated	Furuncle
Yazbek et al. [28]	2019	Quilombo da Fazenda, São Paulo	Myrtaceae (Lythraceae)	*Eugenia uniflora* L.	Pitanga	Leaves; Fruit	Decoction	Bruises
Yazbek et al. [28]	2019	Quilombo da Fazenda, São Paulo	Myrtaceae (Lythraceae)	*Psidium guajava* L.	Goiaba-branca	Leaves	*In natura*	Coagulant
Yazbek et al. [28]	2019	Quilombo da Fazenda, São Paulo	Nyctaginaceae	*Mirabilis jalapa* L.	Maravilha	Leaves	Decoction	Diabetes
Yazbek et al. [28]	2019	Quilombo da Fazenda, São Paulo	Phyllanthaceae	*Phyllanthus niruri* L.	Quebra-pedra-branca	Whole plant	Decoction	Diarrhea; Cough; Sore throat
Yazbek et al. [28]	2019	Quilombo da Fazenda, São Paulo	Piperaceae	*Piper mollicomum* Kunth	Perta-ruão	Leaves	Decoction	Diarrhea
Yazbek et al. [28]	2019	Quilombo da Fazenda, São Paulo	Piperaceae	*Piper scutatum* Yunck	Jaborandi	Root	Heated	Furuncle
Yazbek et al. [28]	2019	Quilombo da Fazenda, São Paulo	Plantaginaceae	*Plantago australis* Lam.	Trançagem/tanchagem	Leaves	Infusion	To prevent kidney stone
Yazbek et al. [28]	2019	Quilombo da Fazenda, São Paulo	Poaceae	*Coix lacryma-jobi* L.	Capiá	Leaves	Maceration	Coagulant; Joint dislocation
Yazbek et al. [28]	2019	Quilombo da Fazenda, São Paulo	Poaceae	*Cymbopogon nardus* (L.) Rendle	Citronela	Leaves	Maceration	Anesthetic
Yazbek et al. [28]	2019	Quilombo da Fazenda, São Paulo	Poaceae	*Saccharum officinarum* L.	Cana	Leaves	Maceration	Coagulant; Diarrhea; Sore throat; Urinary tract infection; To lose weight
Yazbek et al. [28]	2019	Quilombo da Fazenda, São Paulo	Polygonaceae	*Polygonum* sp.	Erva-fogo	Leaves	Decoction	Helps with childbirth
Yazbek et al. [28]	2019	Quilombo da Fazenda, São Paulo	Polygalaceae	*Senega paniculata* (L.) J.F.B.Pastore and J.R.Abbott	Gelol	Whole plant	Maceration	Insect repellent
Yazbek et al. [28]	2019	Quilombo da Fazenda, São Paulo	Rutaceae	*Citrus reticulata* Blanco	Laranja-mixirica	Leaves	Decoction	Antihypertensive
Yazbek et al. [28]	2019	Quilombo da Fazenda, São Paulo	Rutaceae	*Citrus sinensis* (L.) Osbeck	Laranja	Leaves	Maceration	Toothache; Headache
Yazbek et al. [28]	2019	Quilombo da Fazenda, São Paulo	Rutaceae	*Citrus x limon* (L.) Osbeck	Limão	Bark	Decoction	Burning
Yazbek et al. [28]	2019	Quilombo da Fazenda, São Paulo	Rutaceae	*Zanthoxylum rhoifolium* Lam.	Mamica-de-porca	Bark	Decoction	Flu
Yazbek et al. [28]	2019	Quilombo da Fazenda, São Paulo	Solanaceae	*Solanum capsicoides* All.	Arrebenta-cavalo	Fruit	Decoction	Flu
Yazbek et al. [28]	2019	Quilombo da Fazenda, São Paulo	Urticaceae	*Boehmeria caudata* Sw.	Urtiga-branca	Leaves	Decoction	Cough; Sore throat
Yazbek et al. [28]	2019	Quilombo da Fazenda, São Paulo	Urticaceae	*Cecropia glaziovii* Snethl.	Embaúba / Bauibeira	Leaves	Decoction	Clearance; Diabetes
Yazbek et al. [28]	2019	Quilombo da Fazenda, São Paulo	Urticaceae	*Urera baccifera* (L.) Gaudich. *ex* Wedd.	Urtiga-roxa	Leaves	*In natura*	Furuncle
Yazbek et al. [28]	2019	Quilombo da Fazenda, São Paulo	Verbenaceae	*Lippia alba* (Mill.) N.E.Br. *ex* P.Wilson	Melissa, ponta-livre	Leaves	Decoction	Sore throat; Furuncle
Yazbek et al. [28]	2019	Quilombo da Fazenda, São Paulo	Verbenaceae	*Stachytarpheta cayennensis* (Rich.) Vahl	Gervão	Leaves	Decoction; Syrup	Bronchitis
Yazbek et al. [28]	2019	Quilombo da Fazenda, São Paulo	Zingiberaceae	*Curcuma* sp.	Açafrão	Root	*In natura*	Anemia; Bone trauma; Prostate cancer; Scabies
Yazbek et al. [28]	2019	Quilombo da Fazenda, São Paulo	Zingiberaceae	*Hedychium coronarium* J.Koenig	Angélica	Leaves	*In natura*	Antihypertensive; Soothing
Yazbek et al. [28]	2019	Quilombo da Fazenda, São Paulo	Zingiberaceae	*Renealmia petasites* Gagnep.	Picova	Seeds	Infusion	Digestion

## 3. Results 

**Figure 1:**
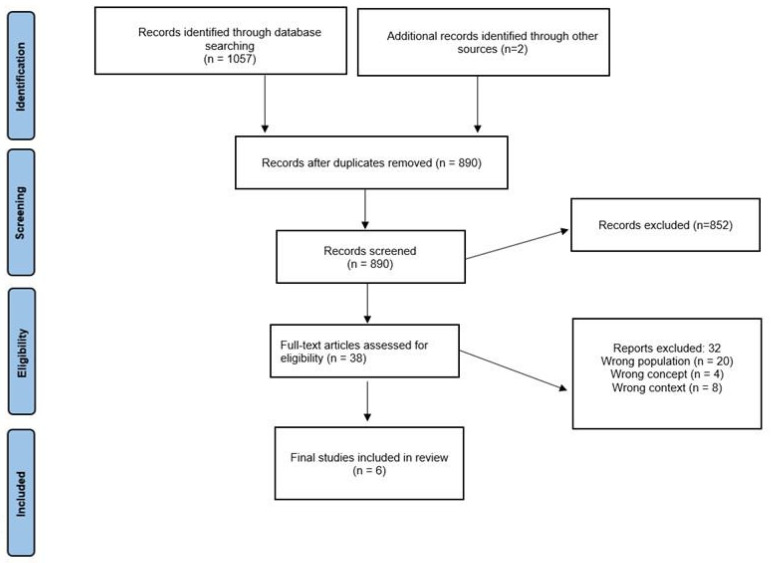
Flowchart detailing the research steps (Source: Authors).

**Figure 2:**
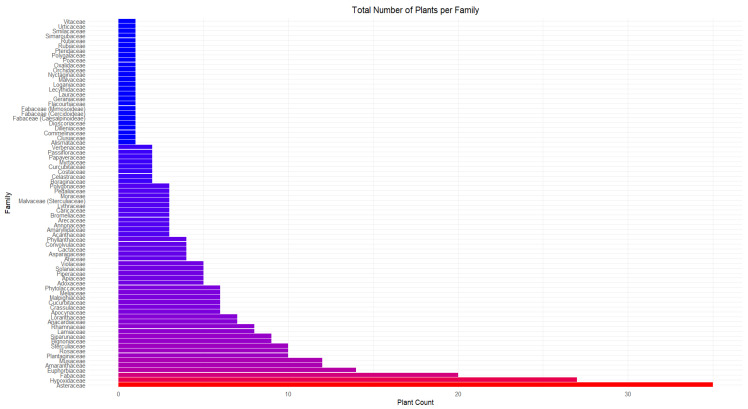
Distribution of plant families in ethnobotanical practices among Quilombola communities.

Using the search criteria, 888 articles were identified. After reviewing the titles and abstracts, 38 studies were selected for further evaluation. Of these, 30 were excluded following a full-text review for not meeting the inclusion criteria, leaving eight studies that were included in the final analysis. An additional two studies were included after a manual search. The details of the research process are illustrated in Flowchart 1 (Figure 1). Across all the selected studies, 297 plants from 80 different families were identified (Figure 2). These plants were used for a wide range of therapeutic purposes. Moreover, the plants were known by various common names, which differed depending on the region where each study was conducted. The most frequently mentioned methods for preparing the harvested plant parts were decoction, alcohol maceration, maceration, and infusion (Table 1). 

The most frequently cited families were Asteraceae Bercht. and J. Presl, with 42 citations [21,22,23,24,26,28], followed by Lamiaceae Martinov [21,22,23,24,25,26,27,28], and Euphorbiaceae Juss [21,22,23,24,28].

The most commonly cited species in the selected articles were *Lippia alba* (Mill.) Britton and P. Wilson [21,22,23,28], *Petiveria alliacea* L. [22,23,26,27], *Momordica charantia* L. [21,22,28], *Ocimum basilicum* L. [21,22,23], *Ruta graveolens* L. [21,22,24,27], and *Senna occidentalis* (L.) Link [21,22,24,26].

The studies included in this research were conducted in communities located in the states of Paraíba, Bahia, Alagoas, Mato Grosso, São Paulo, and Pará. Of the 297 cited species, only 35 are listed in the Brazilian Phytotherapeutic Formulary, a platform that provides information on the correct methods of preparation, indication, and restrictions for the use of medicinal plants in pharmacies, authorized by the National Health Surveillance System [29]. This highlights the importance of research on the medicinal plants used by Quilombola communities, as it contributes to the broader adoption of integrative and complementary health practices.

Table 2 highlights the diverse therapeutic applications of medicinal plants used by Quilombola communities, categorized according to the CIAP-2 system. The most cited usage categories include the digestive system (213 mentions), with common conditions like abdominal pain and liver issues; the respiratory system (179 mentions), dominated by treatments for flu and cough; and general and non-specific conditions (157 mentions), such as malaria, fever, and inflammation. Other significant categories include the nervous system (84 mentions) and the circulatory system (66 mentions), reflecting the communities’ focus on managing headaches, mental health, and cardiovascular conditions. These data underscore the comprehensive knowledge and varied use of medicinal plants across different health-related issues in these communities.

According to the flora and fauna of Brazil, the species *Amburana cearensis* (Allem.) A.C. Smith, *Handroanthus impetiginosus* (Mart. ex DC.) Mattos, *Cuscuta obtusiflora* Kunth, *Piper scutatum* Yunck, and *Handroanthus impetiginosus* (Mart. ex DC.) Mattos are classified as “near threatened”. All other species are classified as “Least Concern” (LC) or have no estimated risk, according to the flora and fauna of Brazil.

## 4. Discussion

For Quilombola communities, the concept of health extends beyond the biological dimension to encompass social, psychological, and political factors. Health is closely tied to their way of life, their connection with nature, and their experiences within their territories. The relationship between the community and nature is therefore a crucial element for the well-being of these populations. This highlights the importance of caring for nature, maintaining direct contact with it, and understanding the interconnectedness between the health of the land and the health of the people [30].

When it comes to accessing health services, it is evident that the basic care available to the Quilombola population, which still heavily relies on the biomedical model focused on curing and medicalization, fails to fully address the specific needs of these communities. Fragmented health care remains prevalent [31]. The use of natural resources for treating illnesses and caring for ecosystems are longstanding traditions among Quilombola communities [23]. These traditions are passed down through generations, though each new generation may introduce new practices and uses. Within the broad ethnobotanical knowledge of these communities, medicinal plants play a key role in the treatment of various diseases, contributing to both environmental conservation and community health [32].

The transmission of knowledge about the use of medicinal plants helps to preserve and strengthen tradition, ensuring it remains alive and contributing to the cultivation of these plants [23]. In this study, it was observed that the same plant species were used for different purposes, depending on the Quilombola community. For instance, in the northeast of Brazil, *Eryngium foetidum* L. and *Foeniculum vulgare* Mill. are used to treat heart attacks and colds, respectively, whereas in the southeast, they are used for snakebites and dysentery. As knowledge is passed down through generations, interpretations of this information and external factors can influence how the therapeutic properties of plants are applied in each region [23].

*Alternanthera brasiliana* (L.) Kuntze was used for treating headaches, while *Annona muricata* L. was indicated for cancer and diabetes in different regions. The use of the same plant species for treating the same disease in various locations suggests their wide distribution across territories and their effectiveness in treating specific conditions [33]. *Lippia alba* is utilized across four different communities with varying therapeutic applications, highlighting the diversity of traditional knowledge, even when the same species is involved. Interestingly, *Momordica charantia* exhibits different applications in geographically distant communities, while its use converges when the communities are in closer proximity. This pattern underscores how both cultural and environmental factors, such as local ecosystems and cultural traditions, influence the choice and preparation of medicinal plants. These findings emphasize the dynamic nature of traditional knowledge, shaped by both shared heritage and local adaptations.

This study, while comprehensive, has some limitations, such as the lack of specific scientific data on the biological and pharmacological properties of many of the plants mentioned by Quilombola communities. Additionally, the variability in documentation and methodologies across the studies reviewed may have limited the comparability of the data. However, this study’s main strength lies in its ability to gather and organize a wide range of traditional knowledge, providing a solid foundation for future ethnopharmacological research and the integration of this knowledge into public health practices. For readers, the study offers the opportunity to appreciate traditional knowledge and understand the connections between culture, health, and biodiversity, while also highlighting the urgent need for further scientific research to deepen our understanding of the plants used by these communities and their potential therapeutic applications. 

In our study, the most commonly used plant parts by the surveyed communities were the leaves. Leaves are preferred for therapeutic preparations because they are easier to collect and more readily available compared to flowers, fruits, and seeds, which may not be accessible year-round [34]. Additionally, traditional communities favor leaves because they believe these parts accumulate active components through photosynthetic pigments, such as alkaloids and tannins [35,36]. Decoction and infusion were the most frequently cited preparation methods in the studies, likely due to the simplicity of using water for preparation [36]. Preparing teas aids in the extraction of active compounds from the plant and helps preserve the solution for longer periods [37].

The study of plants that are endangered or near threatened is crucial for the preservation of native flora, especially in the context of climate change. As ecosystems face increasing pressure from rising temperatures, altered precipitation patterns, and habitat destruction, understanding the status and conservation needs of these species becomes imperative. Protecting these plants not only helps maintain biodiversity but also ensures the survival of traditional knowledge and practices that rely on these species, particularly within communities that have historically depended on them for their health and well-being.

On the other hand, the use of plants based on traditional knowledge holds scientific significance, as it provides direction for research, saving both time and resources [37]. Traditional knowledge helps in selecting plants with potential applications in medicine production. In this context, the discovery of new active molecules often involves a combination of traditional and scientific knowledge [38]. Therefore, the use of medicinal plants by these communities may contribute valuable evidence for the development of pharmaceuticals [39].

## 5. Conclusions

This study highlights the importance of traditional knowledge in the use of medicinal plants among Quilombola communities in Brazil. The diversity of plant species used and the variations in therapeutic applications across different regions demonstrate the richness and adaptability of this knowledge over time. Additionally, the predominant use of leaves in therapeutic preparations and methods such as decoction and infusion reflect the practical wisdom of these communities in extracting active compounds. Preserving and recognizing this knowledge not only contributes to the health and well-being of these communities but also provides a valuable resource for scientific research and the development of integrative health practices. Consequently, this work serves as a foundation for future studies that seek to deepen the understanding and application of Quilombola ethnopharmacological knowledge, fostering cultural conservation and scientific innovation. To achieve this, future research should aim to integrate other disciplines, such as pharmacology, anthropology, environmental science, and history, in an interdisciplinary effort to better understand the evolution of plant use and uncover how traditional knowledge can contribute to sustainable development. Furthermore, it is crucial to ensure the active participation of Quilombola communities in future research, safeguarding their knowledge and ensuring that they directly benefit from any research based on their traditional practices.

## Figures and Tables

**Table 2:** Therapeutic Usage Categories, Based on CIAP-2.

Usage Category (CIAP-2)	Most Cited Therapeutic Indications	Total
A: General and Non-Specific	Malaria (37); Fever (33); Inflammation (23); Cancer (12); Pain (7);	157
D: Digestive System	Abdominal pain (30); Liver (22); Toothache (20); Diarrhea (16); Stomach (15)	213
F: Eyes	Eye inflammation (3); Vision (3); Cataract (1); Conjunctivitis (1); Eye inflammation or pain (1)	10
H: Ears	Ear pain (5)	5
K: Circulatory System	Blood pressure (26); Hypertension (12); High cholesterol (5); Heart attack (5); Antihypertensive (4)	66
L: Musculoskeletal System	Back pain (5); Rheumatism (3); Body pain (2); Leg pain (2); Joint pain (2)	32
N: Nervous System	Headache (42); Mental alteration (10); Migraine (8); Improve learning / nervous breakdown (7); Stroke (6)	84
P: Psychological	Calming (6); Depression (5); Restlessness (3); Sedative (2); To calm (1)	17
R: Respiratory System	Flu (84); Cough (25); Phlegm (13); Sore throat (12); Throat (12)	179
S: Skin	Healing (8); Cuts (7); Bruises (5); Scabies (5); Wounds (4)	44
T: Endocrine, Metabolic, and Nutritional	Diabetes (26); Anemia (22); Detoxifying (5); Anti-inflammatory (1); Lack of appetite (1)	55
U: Urinary System	Kidney stones (7); Kidneys (7); Kidney pain (4); Prevent kidney stones (3); Urethra pain (2); Painful urination (1)	27
W: Pregnancy and Family Planning	Abortifacient (3); Increase contraction (2); Contraceptive/Abortifacient (1)	6
Y: Male Genital System	Prostate cancer (1); Prostate (1)	2
X: Female Genital System (including breast)	Women’s inflammation (13); Menstrual cramps (9); Uterus (7); Ovarian infection (5); Discharge (4)	61
Z: Social Issues	-	0

## Data Availability

No new data were created or analyzed in this study. Data sharing is not applicable to this article.
